# Kinome Profiling Reveals an Interaction Between Jasmonate, Salicylate and Light Control of Hyponastic Petiole Growth in *Arabidopsis thaliana*

**DOI:** 10.1371/journal.pone.0014255

**Published:** 2010-12-08

**Authors:** Tita Ritsema, Martijn van Zanten, Antonio Leon-Reyes, Laurentius A. C. J. Voesenek, Frank F. Millenaar, Corné M. J. Pieterse, Anton J. M. Peeters

**Affiliations:** 1 Plant-Microbe Interactions, Institute of Environmental Biology, Utrecht University, Utrecht, The Netherlands; 2 Plant Ecophysiology, Institute of Environmental Biology, Utrecht University, Utrecht, The Netherlands; 3 Centre for BioSystems Genomics, Wageningen, The Netherlands; Worcester Polytechnic Institute, United States of America

## Abstract

Plants defend themselves against infection by biotic attackers by producing distinct phytohormones. Especially jasmonic acid (JA) and salicylic acid (SA) are well known defense-inducing hormones. Here, the effects of MeJA and SA on the *Arabidopsis thaliana* kinome were monitored using PepChip arrays containing kinase substrate peptides to analyze posttranslational interactions in MeJA and SA signaling pathways and to test if kinome profiling can provide leads to predict posttranslational events in plant signaling. MeJA and SA mediate differential phosphorylation of substrates for many kinase families. Also some plant specific substrates were differentially phosphorylated, including peptides derived from Phytochrome A, and Photosystem II D protein. This indicates that MeJA and SA mediate cross-talk between defense signaling and light responses. We tested the predicted effects of MeJA and SA using light-mediated upward leaf movement (differential petiole growth also called hyponastic growth). We found that MeJA, infestation by the JA-inducing insect herbivore *Pieris rapae*, and SA suppressed low light-induced hyponastic growth. MeJA and SA acted in a synergistic fashion via two (partially) divergent signaling routes. This work demonstrates that kinome profiling using PepChip arrays can be a valuable complementary ∼omics tool to give directions towards predicting behavior of organisms after a given stimulus and can be used to obtain leads for physiological relevant phenomena *in planta.*

## Introduction

Plants defend themselves against a multitude of biotic attackers in several ways. Besides passive barriers, such as wax-layers, needles and trichomes, a variety of induced responses are utilized. These responses rely on defense signaling molecules made by the plant itself. The most prominent of these are salicylic acid (SA), jasmonic acid (JA) and ethylene (ET), and relative levels of these phytohormones depend on the attacker encountered and determine which specific defense response is activated [1]–[4]. In general, JA is synthesized upon insect herbivory, JA and ET predominantly upon necrotrophic pathogen attack, and SA upon infestation with biotrophic pathogens [1]. Not only are SA, JA, and ET produced in different ratios depending on the attacker, they also influence each others action [5]–[8]. For example, SA can repress JA responses [9], [10], and *vice versa*, JA can repress SA responses [2]–[4], [11].

Competition for light is as ubiquitous as encountering attackers and plants are likely to experience the two threats simultaneously in natural situations [12]. When plants encounter competition for light they often induce the so-called shade-avoidance response. Important molecular players in this response are the phytochrome photoreceptors, especially Phytochrome B (PhyB), whereas PhyA is fine-tuning the response [13], [14].

The trade-off between light competition and defense against attackers becomes apparent from several observations. For example, constitutive shade-avoiding mutant plants are more susceptible to herbivore attack than wild type plants [15], [16]. Plants subject to light competition or spectral shade allow a better survival and growth of caterpillars and show reduced JA sensitivity [17]–[19]. The bacterial pathogen *Pseudomonas syringae* induces SA signaling, but the strength of the signal and the response to it depend on light and photoperiod length and on phytochrome-mediated signaling pathways [20], [21]. Moreover, impairment of phytochrome photoreceptors leads to the overproduction of JA [22]. Conversely, several JA mutants have been found in *Arabidopsis thaliana* that are affected in light signaling, e.g. *jar1/fin219* interacts with PhyA [23] and *jin1/myc2* interacts with light-regulated promoters [6], [24]. On top of that a convergence of the PhyA and JA signaling pathways on Jasmonate ZIM-domain1 (JAZ1) was recently discovered [25].

Phosphorylation is an important posttranslational mechanism by which the activity of key enzymes is regulated in response to stimuli. Protein kinases often have a central role in signal transduction pathways and as such mediate many molecular responses within the cells. Studying phosphorylation is generally a cumbersome process, with relatively few tools available. Antibodies recognizing the phosphorylated form of proteins are available for some proteins and can be used for Western blot detection. One way to get an overview of many phosphorylation events is performing mass-spectrometry of phosphorylated peptides, which can be performed in specialized labs [26]–[28]. Kinase substrate arrays can be used for parallel analysis of multiple kinase activities, which is called kinome profiling. These arrays have now been used in mammalian and plant systems to analyze multiple kinase activities in parallel [29]–[32]. Kinase arrays have also been used to study substrates of single kinases, such as MAP-kinase of Arabidopsis and CK2 of barley [33]–[35]. Commercially available PepChip arrays have been used for several purified MAP-kinases from tomato [36]. Kinome profiling on PepChips using Arabidopsis cell extracts obtained from plants infected with an avirulent strain of the bacterial pathogen *Pseudomonas syringae* pv. tomato compared to mock infected plats resulted in the differential labeling of many substrate peptides by kinases present in the cell extracts [37]. In addition, kinome analysis on sucrose-fed Arabidopsis seedlings indicated a role for a Rob small GTPase signal hub in sucrose signaling [38]. In these papers, several differentially phosphorylated consensus peptides were identified, and the involvement of several (types of) kinases was predicted. Surprisingly, kinome profiles showed high similarities among different organisms such as fungi, animals and plants, suggesting the a limited kinase substrate variability in eukaryotes [29]. The observed phosphorylation of the photoreceptor PhyA by an animal-derived Protein Kinase A (PKA) – a class of kinases still controversial in plants – is in line with this idea [39].

To i) analyze posttranslational interactions in MeJA and SA signaling pathways and ii) to test if kinome profiling can be used to predict biologically relevant posttranslational events in plant signaling and behavior at the whole plant level, we profiled the kinome of MeJA- and SA-treated plants. Several substrate peptides were significantly differentially phosphorylated, including plant-specific substrates derived from the photoreceptor protein PhytochromeA (PhyA) and Photosystem II D proteins (PS II D). Using PhyA-dependent low light-induced upward leaf movements (hyponastic growth) as bio-assay [40], [41], we studied the predicted interaction between MeJA signaling and (PhyA-dependent) light signaling. Notably, induction of hyponastic growth in low light was repressed by JA, and by SA, and this phenomenon was fully mimicked by application of the JA-inducing insect herbivore *Pieris rapae*. These results suggest that kinome profiling can complement available tools for genomics and proteomics research as it may lead to prediction of biologically relevant responses to external stimuli.

## Results

### 1. Kinome profiling of MeJA and SA treated plants using PepChip arrays

To obtain the kinome fingerprint of plants treated with defense related hormones, Arabidopsis accession Columbia-0 (Col-0) seedlings were incubated for 1 h and 6 h with MeJA, SA or a combination of the two (SA/MeJA). Specificity of MeJA and SA responses was confirmed 6 h post-incubation by quantification of transcript accumulation of the inducible marker genes *PDF1.2* (MeJA) and *PR1* (SA), by quantitative RT-PCR (data not shown). As expected, *PR1* expression was up regulated by SA and SA/MeJA, whereas *PDF1.2* expression was up regulated by MeJA and down regulated in the SA/MeJA combination treatment, compared to JA-treatment alone [3], [9], [42], [43].

A total of twelve PepChips containing 1178 consensus peptides (in duplicate) were incubated with extracts obtained from three independently grown biological replicates treated for one hour with either control solution, MeJA, SA, or SA/MeJA (Dataset S1). Several peptides showed differences in intensity between treatments, indicating differential phosphorylation (Figure S1). The majority of phosphorylated peptides was phosphorylated to similar levels in all treatments, and supposedly represent general peptides that detect kinase activities involved in basic cellular functioning [29].

**Figure 1:**
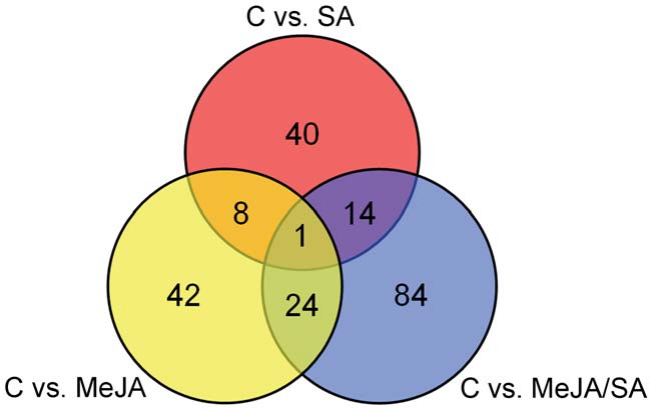
Venn diagram of differentially phosphorylated substrates upon treatment (MeJA, SA or SA/MeJA) versus control (C). The individual differentially phosphorylated peptides are shown in Table S1; peptides that are differentially phosphorylated after two or more treatments are represented in the table in subsequent rows. Of the nine (8+1) spots, differentially phosphorylated by both MeJA and SA, all were changed in the same direction, this was also observed for those that were differentially phosphorylated by SA/MeJA and overlap with SA or MeJA.

Compared to control treatment, we identified 75 significantly differentially phosphorylated peptides upon MeJA treatment, 63 upon SA treatment and 123 upon the combination treatment (for a complete list see Table S1). Surprisingly, many spots were uniquely differentially phosphorylated by either SA, MeJA, or SA/MeJA, and relatively little overlap was found (Figure 1). This indicates that signaling following SA/MeJA treatment is not simply the sum of the signaling that occurs after SA and MeJA single treatments.

### 2. General description of differentially phosphorylated peptides by plant defense hormones

The PepChip analysis showed that 37 substrate residues annotated to detect Tyrosine (Tyr) phosphorylation were differentially phosphorylated; generally the phosphorylation-level is increased upon each of the treatments (Table SI). Although tyrosine phosphorylation in plants is debated in general, and tyrosine receptors are controversial, examples of tyrosine phosphorylation are present (Text S1). Also Protein Kinase A (PKA) activity in plants is controversial. The artificial substrate Kemptide (RRASLG) is generally used to monitor PKA activity in animal tissues and is shown to be a substrate of Arabidopsis AGC-kinase 2 [44]. Several Kemptide analogues showed increased phosphorylation after the treatments (12 out of 15; Table SI). Two exceptions were the more divergent peptides RRAASVA and RRASS, which had decreased phosphorylation. Other differentially phosphorylated targets of PKA and PKC, together belonging to the AGC-kinases, were generally increased (20 out of 27; Table SI). Substrates of the Casein Kinase II (CKII) showed generally decreased phosphorylation after different treatments (13 out of 15).

Cell Division Cycle 2 (CDC2) is represented on the PepChip by the peptide GEGTYGVVY, which is identical in the Arabidopsis CDC2 orthologue (At3g48750). On this peptide consensus motifs for the inhibitory kinases WEE and MYT (also found in Arabidopsis) are present [45], [46]. MeJA treatment increased phosphorylation of the peptide, which suggests inhibition of CDC2 activity. On the other hand, several CDC2 target substrates were higher phosphorylated after SA and SA/MeJA treatment. One CDC2-annotated peptide was also higher phosphorylated after JA, but another showed lower phosphorylation upon MeJA treatment.

Two substrate peptides present in Pyruvate Dehydrogenase (PDH; DPGTSYRTR and YSGHSMSDP) had increased phosphorylation after MeJA treatment (one also after SA/MeJA) indicating activation of PDH-kinase. Both peptides are highly similar to peptide sequences in the orthologous Arabidopsis PDHs (At1g24180 and At1g59900). For a more elaborate presentation of differentially phosphorylated peptides, see Text S1.

### 3. Kinome and transcriptome analysis reveals regulation of plant specific processes by jasmonate

Ten PepChip peptide substrates are derived from plant-specific proteins (Table 1). None of the plant-specific peptides showed significantly differential phosphorylation after SA treatment. However, four were differentially phosphorylated after MeJA treatment: an aquaporin water channel derived peptide, a Phosphoenol Pyruvate Carboxylase (PEP carboxylase) derived peptide, a Photosystem II D protein (PSII D) derived peptide and the photoreceptor phytochrome A (PhyA) derived peptide (Table 1). Additionally, peptides of PhyA, PSII D and a Sucrose Synthase showed enhanced phosphorylation after combined SA/MeJA treatment (Table 1). This indicates that the proteins from which these peptides are derived are post-translational regulated during MeJA and SA/MeJA signaling.

**Table 1:** Plant-specific peptide-substrates differentially phosphorylated after MeJA and SA/MeJA treatment.

compare	p-value	P	motif	P-site	kinase	Swiss-prot reference	substrate
C-MeJA	0.042	▾	EKHHSIDAQ	S-15	PEPCk	SWISS;P04711; CAP1_MAIZE	PEP Carboxylase
C-JA	0.023	▴	TKSASFLKG	S-262	CDPK	SWISS; P08995; NO26_SOYBN	aquaporin
C-SA/MeJA	0.037	▴	TIAVG	T-1	STN8	SWISS;P06005; PSBD_SPIOL	photosystem II D2 protein
C-JA	0.043	▴	TIAVG	T-1	STN8	SWISS;P06005; PSBD_SPIOL	photosystem II D2 protein
C-SA/MeJA	0.011	▴	KREASLDNQ	S-598	PKA	SWISS;P06593; PHY3_AVESA	Phytochrome-A
C-JA	0.024	▴	KREASLDNQ	S-598	PKA	SWISS;P06593; PHY3_AVESA	Phytochrome-A
C-SA/MeJA	0.047	▴	SRLHSVRER	S-15		SWISS;P49036; SUS2_MAIZE	sucrose synthase

***Footnote:*** Plant-specific peptide-substrates are shown that are increased (▴) or decreased (▾) phosphoryated (P) compared to control (C). Peptide sequence (motif), phosphorylation site (P-Site), predicted kinase to phosphorylate the target peptide, and target protein from which the peptide sequence is derived (SWISS annotation) are indicated as presented in supporting PepScan documentation (www.pepscanpresto.com). Significant differences in phosphorylation (p<0.05) compared to control (C) were determined using a Student's *t*-Test on three replicates of plants grown at different moments in time.

The MeJA control of photosynthesis and light-related factors as suggested by kinomics is supported by transcriptomics data. Re-evaluation of previously published microarray data [1], [47] by MapMan analysis revealed that the significantly (p<0.05) altered bins in plants incubated for 1 h with MeJA, were ‘stress’, ‘signaling’ and ‘photosynthesis’ (PS; Table 2). After 3 h and 6 h incubation several other bins became significantly altered. PS and stress were the only ones that remained significantly different at all tested time points. The ‘PS’ bin contains three sub-bins (Table S2). At all time points the ‘light reaction’ bin (1.1) was significantly different. Within this sub-bin, ‘PSII’ (bin 1.1.1) was consistently significantly down regulated after MeJA treatment. In addition, ‘light signaling’ (bin 30.11, harboring among others the phytochrome photoreceptors) was also significantly down regulated after 3 h (p = 1.15E-2) and 6 h (p = 1.01E-3) of MeJA treatment. Genes involved in SA, ET and JA metabolism are grouped in bin 17 (‘hormone metabolism’) which was significantly different at 3 h and 6 h after MeJA treatment (Table S3). As expected, MeJA induced several JA biosynthetic genes [48] (e.g. *LOX2*, data not shown).

**Table 2:** Gene representation of functional classes differentially expressed upon MeJA treatment calculated by MapMan.

bin	name	elements	p-value 1 h	p-value 3 h	p-value 6 h
1	PS	174	**2.53E-04**	**1.51E-12**	**7.18E-06**
2	major CHO metabolism	79	9.61E-01	1.01E-01	6.82E-02
3	minor CHO metabolism	95	4.74E-01	6.33E-01	9.74E-01
4	glycolysis	55	9.67E-01	4.44E-01	**3.22E-03**
5	fermentation	13	2.41E-01	**8.92E-04**	**4.81E-03**
6	gluconeogenese/glyoxylate cycle	6	8.32E-01	9.88E-01	9.69E-01
7	OPP	29	5.70E-01	4.98E-01	3.19E-01
8	TCA/org. transformation	56	5.68E-01	6.13E-01	**2.88E-02**
9	mitochondrial electron transp./ATP synth.	91	3.12E-01	**1.17E-02**	**2.25E-06**
10	cell wall	271	8.31E-01	1.45E-01	**1.44E-02**
11	lipid metabolism	294	1.76E-01	4.51E-01	1.30E-01
12	N-metabolism	23	7.98E-01	4.41E-01	2.43E-01
13	amino acid metabolism	271	8.81E-01	2.95E-01	**3.61E-02**
14	S-assimilation	13	8.17E-02	9.88E-01	9.13E-01
15	metal handling	56	5.99E-01	7.25E-01	8.06E-01
16	secondary metabolism	255	4.74E-01	5.19E-01	9.74E-01
17	hormone metabolism	362	1.22E-01	**9.26E-03**	**2.60E-06**
18	Co-factor and vitamine metabolism	41	8.04E-01	9.89E-01	3.79E-01
19	tetrapyrrole synthesis	41	1.19E-01	6.89E-01	5.28E-01
20	stress	501	**7.39E-06**	**1.27E-03**	**1.16E-05**
21	redox.regulation	155	7.95E-01	1.66E-01	**1.44E-03**
22	polyamine metabolism	13	9.71E-01	8.04E-01	4.03E-01
23	nucleotide metabolism	122	5.57E-01	3.42E-01	6.51E-02
24	Biodegradation of Xenobiotics	17	5.74E-01	6.70E-01	9.13E-01
25	C1-metabolism	25	4.66E-01	6.45E-01	7.99E-01
26	misc	710	6.13E-02	**1.50E-06**	**7.18E-06**
27	RNA	1693	5.33E-01	**2.63E-02**	**2.92E-07**
28	DNA	339	9.67E-01	6.47E-01	7.75E-01
29	protein	2159	3.69E-01	5.44E-01	7.88E-02
30	signalling	753	**5.30E-03**	9.58E-01	8.71E-02
31	cell	444	6.11E-01	7.87E-01	9.23E-01
33	development	303	8.81E-01	4.79E-01	3.94E-01
34	transport	627	3.93E-01	1.28E-01	8.06E-01
35	not assigned	5362	**1.49E-03**	1.07E-01	5.88E-01

***Footnote*** **:** Bins show clusters of genes (elements) designated to a bin, and the probability that the genes within the bin are differentially expressed upon 1 h, 3 h and 6 h MeJA treatment. Significant probabilities (p<0.05) are designated in bold.

These transcriptomics data corroborate our PepChip results, suggesting that MeJA influences light-related factors. It seems that this occurs at both the transcriptional and posttranslational level. Together, these results predict interaction between the JA pathway and light signaling, which is in accordance with recently published data [25].

### 4. Jasmonate controls low light-induced differential hyponastic petiole growth

To confirm the predicted interaction between JA and SA/MeJA signaling and light signaling, we tested interference of MeJA with low light intensity-induced upward leaf movement (hyponastic growth), as this response is controlled by both PhyA and photosynthesis-related signals [40], [41], [49].

**Figure 2:**
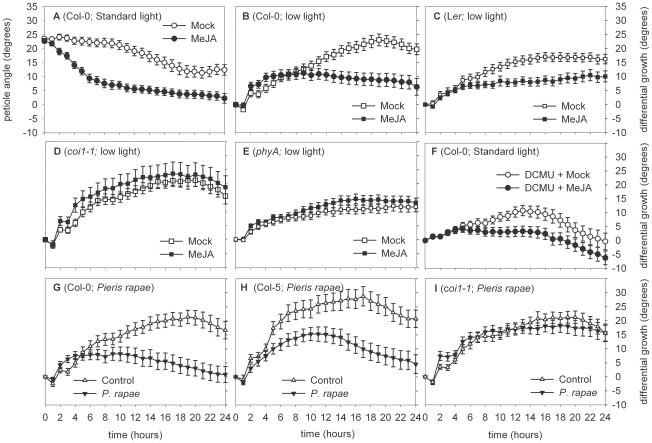
MeJA effects on light controlled leaf movements. (A–F) effect of (1 h) pre-treatment with 100 µM MeJA (closed circles), compared to mock (open circles), or (G–I) infestation with *Pieris rapae* (closed triangles) caterpillars compared to control (open triangles), on Arabidopsis petiole angles. Plants were (A,F) in standard light (200 µmol m^−2^ s^−1^) or (B–E,G–I): treated with low light (200 µmol m^−2^ s^−1^ to 15 µmol m^−2^ s^−1^). (A,B,G) Col-0 wild type; (F) Col-0 wild type treated with 10 µM 3-(3,4-dichlorphenyl)-1,1-dimethylurea (DCMU); (D,I) *coi1-1*; (C) Landsberg *erecta* (L*er*) wild type; (E) *phytochrome A* (*phyA-201*) mutant (in L*er*); (H) Col-5 wild type. Note that the Y-axis in panel (A) is different from the other panels (b–i) as here the angle relative to the horizontal is depicted, as opposed to (B–I) which shows pair-wise subtracted values per time point for the corresponding response in control conditions. Error bars represent SE, n>12 petioles.

**Figure 3:**
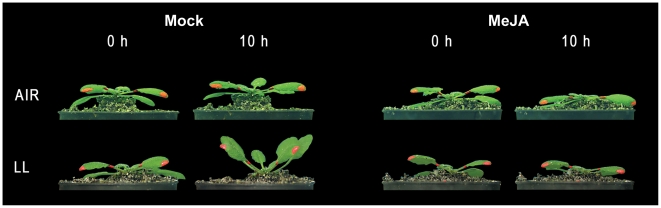
Typical effects of MeJA on leaf movement. Hyponastic growth phenotype of Arabidopsis Col-0 in control conditions or after 10 h low light (LL; 200 µmol m^−2^ s^−1^ to 20 µmol m^−2^ s^−1^) treatment in the presence of MeJA, or a mock solution. Note that the petiole-lamina border was painted to facilitate time-lapse measurement of leaf angle kinetics.

First, we assessed if MeJA had an effect on petiole angles under standard light conditions (200 µmol m^−2^ s^−1^). Vegetative Arabidopsis Col-0 plants were soil drenched with 100 µM MeJA or mock (control) solution 1 h prior to the start of observation. Kinetics of petiole movement was subsequently monitored during the next 24 h using a time-lapse camera-setup [40], [50]. In control conditions, petiole angles slightly decreased over time due to aging and diurnal effects [40]. This decrease was enhanced by MeJA (Figure 2A; Figure 3).

Hyponastic growth, as induced by low light-intensity (reduction of the photosynthetic active radiation from 200- to 15 µmol m^−2^ s^−1^), was repressed by MeJA in both Col-0 and Landsberg *erecta* (L*er*) (Figure 2B, C; Figure 3). Initially, leaf inclination keeps track of non-MeJA treated plants, but after ∼8 h, the MeJA-treated plants reached equilibrium, whereas petiole angles in non-MeJA treated plants steadily increased. As expected, MeJA did not affect low light-induced hyponastic growth in the JA-insensitive *coronatine insensitive1-1* receptor mutant (*coi1-1* in Col-5) [51] (Figure 2D). This confirms that JA signaling is involved in the repression of low light-induced hyponastic growth. MeJA did also not influence hyponastic growth induced by low light in the *phya* mutant background (in L*er*; Figure 2E), indicating the involvement of PhyA in the interference of JA signaling with this response.

To investigate the involvement of photosynthesis signaling in the observed phenomenon, we repressed photosynthetic electron transport by 3-(3,4-dichlorphenyl)-1, 1-dimethylurea (DCMU). This results in a hyponastic growth phenotype under standard light conditions and is indicative for the involvement of photosynthesis-related signals in the control of petiole angles [41]. Application of MeJA to DCMU treated plants suppressed the DCMU-induced hyponastic response (Figure 2F), indicating that MeJA interferes with signals originating from reduced photosynthesis.

Tissue chewing caterpillars such as larvae of the small cabbage white butterfly *Pieris rapae*, induce JA accumulation in Arabidopsis [1]. *P. rapae* was applied to Col-0 plants 20 h before the switch to low light. Severe damage (of each leaf ∼50% was eaten) was visible at the start of the low light treatment (data not shown) and was comparable in Col-0 and Col-5 plants that lack trichomes [52]. Hyponastic growth was repressed by *P. rapae* in a similar manner as was seen for MeJA (Figure 2B, G, H). When *P. rapae* infested JA-signaling deficient *coi1-1* plants, the hyponastic response to low light conditions was not influenced (Figure 2I). This suggests that the reduction of hyponasty as seen after *P. rapae* infestation is a direct consequence of the JA response induced by the caterpillar and is not resulting from physical damage.

Together, the data on hyponastic growth support the kinome- and transctiptome-derived predictions that MeJA interferes with light-signalling.

### 5. Salicylate suppresses hyponastic growth to low light synergistically to jasmonate

The PepChip data showed no significant changes in phosphorylation of plant-specific substrate peptides after SA treatment. However, a non-significant increase in phosphorylation of the PhyA and PSII D peptides was observed (data not shown) and the combined treatment (SA/MeJA) resulted in a significant increase in phosphorylation of both peptides (Table 1).

**Figure 4:**
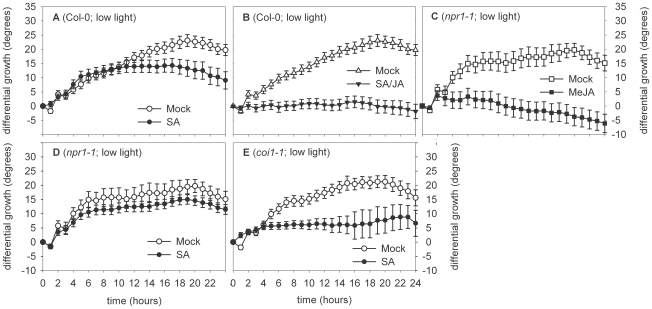
SA and SA/MeJA effects on light-controlled leaf movements. (A,D,E) Effect of 1 h pre-treatment with 1 mM SA (closed circles), (C) 100 µM MeJA (closed squares) and (b) the combination SA + MeJA (SA/MeJA, 100 µM, closed squares) compared to mock (open symbols), on Arabidopsis petiole angles in low light. (A,B) Col-0 wild type; (C,D) *npr1-1*; (E) *coi1-1*. Values were pair-wise subtracted per time point for the corresponding response in control conditions. Error bars represent SE, n>12 petioles.

Application of SA to Arabidopsis plants suppressed low light-induced hyponastic growth (Figure 4A), similar to MeJA treatment. The combination SA/MeJA showed synergism, since this resulted in complete abolishment of the hyponastic growth response (Figure 4B). The SA-insensitive *non-expresser of PR genes 1* (*npr1-1*) mutant showed a normal low-light induced hyponastic growth response in the presence of SA (Figure 4D). This indicates that the repression by SA depends on intact SA signaling routes. MeJA repressed low light-induced hyponastic growth in *npr1-1* (Figure 4C). *Vice versa*, SA application to the JA-signaling mutant *coi1-1* led to repression of low light-induced hyponastic growth (Figure 4E).

Together, these data suggest that besides JA, also SA is a modulator of light intensity-dependent differential petiole growth. Furthermore, MeJA and SA appear to act additive and exert their function via at least partially different JA- and SA-specific signaling routes.

## Discussion

### 1. Profiling of JA-directed differential phosphorylation

PepChips can be used to identify differential kinase activity, and is a helpful tool to study post-translational regulation in signal transduction pathways [29], [30], [32], [37], [38], [53]. To this aim, we profiled the kinome of Arabidopsis plants treated with the defense inducing hormones MeJA and SA. We identified significant changes in the phosphorylation of several substrate peptides linked to the activity of protein kinases (See Text S1; Table S1). Among the general differentially phosphorylated substrate residues were those annotated to detect Tyrosine (Tyr) phosphorylation [29], [54], [55]. The artificial substrate Kemptide is used to monitor activity of a subgroup of AGC-kinases [44], CKII substrates, Cell Division Cycle 2 (CDC2) kinase and some of its substrates (Text S1; Table S1). CKII is involved in general cellular processes such as circadian rhythm and cells cycle progression [56], [57]. The CDC2-related differences in phosphorylation activity point towards inhibition of cell cycle progression by JA, which was indeed observed before [58], [59].

In addition, our analysis revealed differential phosphorylation of plant-specific peptide substrates (Table 1). These included substrates from the plant specific aquaporin proteins, belonging to the major intrinsic protein super family and known for regulating water status and processes such as petal opening and cell elongation [60]–[62]. MeJA directed enhanced phosphorylation, which is indicative for activation of the water channel [61], [63].

The reduced phosphorylation observed after MeJA treatment of a peptide derived from phosphoenol pyruvate carboxylase (PEP carboxylase) suggests decreased activity [64] and consequently reduced replenishment of TCA/Krebs cycle intermediates.

Light reactions of photosynthesis are mediated by PSI and PSII. The PSII reaction center contains two D proteins, which are phosphorylated in a circadian fashion by the specific kinase STN8 [65], [66].

PhyA, one of the five red and far-red light perceiving Ser/Thr kinases (PhyA-PhyE), regulates the transcription of *psbD*, encoding the PSII D2 protein [67]. Interestingly, from our PepChips, PhyA is suggested to be phosphorylated upon MeJA and SA/MeJA treatment. It should however be noted, that the actual peptide (KREASLDNQ) on the PepChip is derived from oat (*Avena sativa*) PhyA and is not as such present in Arabidopsis (Table 1). The PhyA protein is light labile in white light [68], [69] and enhanced phosphorylation of residues Ser7 and Ser17 in these conditions negatively influences protein stability [70], [71]. Similarly, decreased autophosphorylation resulted in enhanced stability of the PhyA protein in light in the natural *phyA* mutant accession Lm-2 [72]. Because the residues Ser7 and Ser17 are not represented on the PepChip, we cannot study these phosphorylations and can therefore not predict if PhyA stability would be altered upon MeJA application. That this might be the case is suggested by the JA requirement, recently identified by Riemann *et al.* [73], for photodestruction of PhyA upon (high) light stimulus.

### 2. JA and SA hormones influence differential hyponastic petiole growth

We demonstrated that MeJA decreases hyponastic growth of petioles in response to reduced light intensity. Re-analysis of genome-wide transcript profiling using MapMan software showed that ‘PS and signaling’ bins (Table 2, Table S2), including the phytochromes, have an altered transcription (overall a down-regulation) upon MeJA treatment. This is in accordance with earlier observations that MeJA can inhibit light-inducible and photosynthesis-related genes [22] and indicates that the MeJA effect not only acts on the post-translational level but also affects light sensitivity in a indirect manner by preventing transcription of photosynthesis-related genes.

Recently, an interesting study was published showing that JA signaling and light signaling converges at the level of JAZ stability [25]. JAZ breakdown by COI1 was already identified as an essential component in JA signaling [74], [75]. Our results demonstrate that MeJA suppresses low light-induced hyponastic growth in a PhyA- and COI1-dependent manner (Figure 2, Figure 3), indicating that JAZ degradation likely is also involved in this.

High light conditions increase the relative level of phosphorylated PSII D proteins [65]. The prediction that MeJA increases phosphorylation levels of PSII D indicates that MeJA brings this protein in a ‘high light’ state. It is to our knowledge unknown what the effect of phosphorylation of PSII D proteins is, but a role in inhibition of photosynthesis is suggested [65]. Interestingly, we demonstrated that MeJA interferes with signals originating from reduced photosynthesis towards the induction of hyponastic growth. It would be interesting to know if photosynthesis-related signals also affect JAZ stability.

SA treatment was also able to repress low light-induced hyponastic growth in a NPR1-dependent manner (Figure 4). The combination treatment of MeJA and SA abolished the hyponastic response completely, suggesting a synergistic effect of both hormones in interfering with light signaling. Differential pathways are proposed for MeJA and SA interference, since COI1 is required only for the effect of MeJA and NPR1 only for the effect of SA.

In conclusion, this paper demonstrates that PepChip arrays can provide leads to detect physiologically relevant phenomena *in planta*. However, with only 10 plant specific peptides on the current generation PepChips chances doing so for other than light-signaling events will be limited. Moreover, in most cases it is not yet known which kinase is responsible for certain phosphorylation events and it is also not always known whether phosphorylation results in activation or inhibition of the activity of the target.

Development of dedicated PepChip arrays containing more plant specific substrates would be a valuable addition to established ∼omics tools such as transcriptomics, proteomics and metabolomics for plant research. However, a better understanding of kinases and phosphorylation events in plants is required before the use of PepChips to detect and predict new pathways and interconnections of existing pathways can come to full bloom.

## Materials and Methods

### 1. Pepchip-, Micro-array-, and RT-PCR analysis

For kinome profiling, *Arabidopsis thaliana* Columbia-0 (Col-0) plants were grown for 16-17 d on cellulose filters, on agar plates (ø 10 cm) containing ½ Murashige & Skoog medium, at 20°C, 9 h photoperiod of 70 µmol m^−2^ s^−1^ photosynthetic active radiation (PAR). Individual biological replicates were grown at different moments in time.

One hour after the start of the photoperiod, 1 ml 100 µM MeJA (Serva, Heidelberg, Germany) in 0.1% ethanol or mock solution with 0.1% ethanol only, were applied to the filters. Plants were harvested 1 h and 6 h after this incubation, plants were harvested and snap-frozen in liquid N_2_ and stored at −80°C.

For real time Reverse Transcriptase-PCR (RT-PCR), 80 mg (FW) Col-0 seedlings were used for RNA isolation according to the Invisorb Spin Plant RNA mini kit (Invitek/Westburg, Leusden, the Netherands). The absence of genomic DNA was checked by PCR, using *PDF1.2* (At5g44420) primers (5′-TCACCCTTATCTTCGCTGCTC-3′, 5′-TGTAACAACAACGGGAAAATAAACA-3′) that are intron overspanning. cDNA was made using SuperScript III (Invitrogen, Breda, the Netherlands) and real time RT-PCR performed with SYBRgreen (Biorad, Veenendaal, the Netherlands) on an iCycler (Biorad). The following primer combinations were used: *PDF1.2*; 5′-CGAGAAGCCAAGTGGGACAT-3′, 5′-TCCATGTTTGGCTCCTTCAA-3′; *PR1* (At2g14610); 5′-CTCGGAGCTACGCAGAACAACT-3′, 5′-TTCTCGCTAACCCACATGTTCA-3′ and Ubiquitin10 (At5g05320) 5′-GGCCTTGTATAATCCCTGATGAATAAG-3′, 5′-AAAGAGATAACAGGAACGGAAACATAGT-3′ as control. Kinome profiling was performed essentially as described before [37]. Lysate was made from 100 mg (FW) homogenized plants in 200 µl lysisbuffer (20 mM Tris-HCl, pH 7.5, 150 mM NaCl, 1 mM EDTA, 1 mM EGTA, 1% Triton X-100, 2.5 mM Na_4_P_2_O_7_, 1 mM β-glycerophosphate, 1 mM Na_3_VO_4_, 1 mM NaF, 1 µg ml^−1^ leupeptin, 1 µg ml^−1^ aprotinin and 1 mM PMSF added fresh from a stock of 100 mM in isopropanol). The lysate was kept on ice for 5 to 10 min and debris was pelleted by centrifugation at 4°C. Supernatants were filtered through a 0.2 µm mesh filter (Nanosep MF, Pall, Port Washington, NY, USA) to remove particles.

Cell lysates were activated by the addition of 12.25 µl fresh activation mix (10 ml 50% glycerol, 0.15 ml 100 mM ATP, 0.6 ml 1 M MgCl_2_, 0.1 ml 3% Brij-35, 0.3 ml 5 mg ml^−1^ BSA) to 50 µl of lysate. PepChip Kinase slides (PepScan Systems, Lelystad, the Netherlands) were incubated with the lysate plus activation mix and 3 µl radioactive ATP (3 µCi γ-[^33^P]ATP, specific activity 1000–3000 Ci mmol^−1^, GE Healthcare/Amersham, Buckinghamshire, UK). After incubation for 2 h at 30°C in 100% relative humidity, the slides were washed twice in PBS with 0.05% Tween20, twice in 0.5 M NaCl and twice in milliQ-purified water. Each wash step was performed for 5 min, after which they were placed in a phosphorimager cassette with an imager screen. After 7 d exposure, radioactivity was quantified using a Phosphorimager with QuantityOne software (Biorad). Slides were analyzed using the freeware tool ScanAlyze http://rana.lbl.gov/EisenSoftware.htm [76]. All ScanAlyze analysed data generated in this work data is available via dataset S1.

Spot intensities were determined and averaged between the two replicates present on one PepChip before intensities of control and JA-treated plants were compared individually for the 3 experiments. Significant differences were determined using a Student's *t*-Test (Table S1). The peptide annotation used was derived from the PepScan documentation (www.pepscanpresto.com).

Micro-array studies were previously described by De Vos *et al.* [1] and Pozo *et al.* [47]. Plants were treated with 50 µM MeJA for 1 h, 3 h, or 6 h and cDNA synthesized from leaf-derived mRNA was spotted on ATH1-Affymetrix microarrays. The datasets are available via http://affymetrix.arabidopsis.info; *NASCArrays Experiment Reference Numbers*: NASCARRAYS-463 and -330. In our analysis, spots were only considered for MapMan analysis when the expression value was above 40 for at least one of the time points. Log2 transformations of the expression ratio's (compared to control plants) were calculated and fed into the MapMan program (version 1.8.0; http://gabi.rzpd.de/projects/MapMan/) [77]. Statistical analysis was performed using the Wilcoxon Rank Sum test and is Benjamini-Hochberg corrected.

### 2. Hyponastic growth measurements

Plants used for hyponastic growth measurements were grown as previously described [40] on a mixture of potting soil and perlite (1∶2) at: 20°C, 70% (v/v) relative humidity and 9 h photoperiod of 200 µmol m^−2^ s^−1^ PAR. Pots were automatically saturated daily with tap water at the start of the photoperiod, until three days before the start of the experiment.

Col-0 (N1092), Col-5 (N1644), Landsberg *erecta* (L*er*; NW20) and *phyA-201* (N6219) [78] were obtained from the Nottingham Arabidopsis Stock Center (ID numbers are shown between brackets), *npr1*-*1* is described in Cao *et al.* [79], *coi1-1* is from the lab of J. Turner [80]. Because the mutant *coi1-1* is male-sterile, this line was propagated in a heterozygous state. Before use, *coi1-1* plants were grown on ½ Murashige & Skoog medium containing 1% sucrose and 0.01 mM MeJA. JA-resistant plants were scored by their increased root length and transferred to the above-described soil mixture.

Approximately 5 weeks-old plants, at developmental stage 1.09 to 1.12 according to Boyes *et al.* [81] were used. All treatments started (t = 0) 1.5 h after the start of the photoperiod. Low light treatment consisted of reduction of the light intensities (PAR) to 15 µmol m^−2^ s^−1^, and was accomplished by switching off several lamps in the growth-cabinet, and by shading the plants with spectrally neutral shade cloth. The spectral quality and quantity was checked with a LICOR-1800 spectro-radiometer (LI-COR, Lincoln, NE, USA).

Plants subjected for treatment were withhold water three days prior to the experiment. MeJA was dissolved in ethanol and subsequently diluted in milliQ-purified water to a concentration of 100 µM MeJA; 1 ‰ ethanol. SA (Duchefa, Haarlem, the Netherlands) was dissolved in water to a concentration of 1 mM. Ethanol was added (1‰) to the SA solution to allow comparison with MeJA experiments. Mock solutions only contained ethanol (1‰). The control solution for the *P. rapae* experiments was milliQ-purified water. All solutions were added to the soil until saturation 1 h prior to the start of the experiment (0.5 h after start of the photoperiod). Photosynthesis was inhibited by spraying the plants 1 h prior to the start of the experiment once with 10 µM 3-(3,4-dichlorophenyl)-1,1-dimethylurea (DCMU) in 0.1% tween20 and 0.01% ethanol. The control plants were sprayed with a solution containing 0.1% tween20 and 0.01% ethanol only.

*Pieris rapae* larvae were raised essentially as described in De Vos *et al*. [1]. Larvae from the 3 first-instar stage were applied to each plant 20 h before the start of the treatment with a small paintbrush.

Angle kinetics experiments were conducted using automated time-lapse photography as described before [40], [41], [50], [82]. To enable continuous photography, no dark period was included in the 24 h experimental period. Additionally, petiole/lamina junction was marked with orange paint (Decofin Universal, Apeldoorn, the Netherlands). Plants were transferred to the experimental setup singly, in glass cuvettes, one day before the experiment to allow acclimatization. These preparations did not influence the response of the petiole (data not shown). The light regime in the cuvettes was the same as during the growth period until the experiment started.

Digital photographs were taken every 10 min of two petioles per plant that were approximately 1 cm in length. Petiole angles were measured between the orange painted point at the petiole/lamina junction and a fixed basal point of the petiole, compared to the horizontal, by using KS400 (Version 3.0) software package (Carl Zeiss Vision, Hallbergmoos, Germany) and a customized macro developed in house. To take into account the changes in angle of control plants during the course of the experiments, we did a pair wise subtraction, which is the difference between the angles of treated and control plants for each time point [82]. This corrects the treatment-induced petiole movement for circadian and/or diurnal variations. Calculation of the new standard error for the differential response was performed by taking the square root from the summation of the two squared standard errors.

## Supporting Information

Dataset S1 ScanAlyze-analysed data generated in this study.
(3.38 MB XLS)

Table S1 Significant differential phosphorylated peptides after MeJA, SA or SA/MeJA treatment compared to control.
(0.48 MB DOC)

Table S2 Gene representation of functional classes differentially expressed upon MeJA treatment in the ‘photosynthesis’ sub-bin calculated by MAPMAN.
(0.04 MB DOC)

Table S3 Gene representation of functional classes differentially expressed upon MeJA treatment in the MAPMAN ‘hormone metabolism’ sub-bin.
(0.05 MB DOC)

Figure S1 Typical PepChip autoradiogram.
(10.43 MB TIF)

Text S1 General analysis of observed kinome profiles.
(0.05 MB DOC)
