# Supplementary material for: Kinome Profiling Reveals an Interaction Between Jasmonate, Salicylate and Light Control of Hyponastic Petiole Growth in Arabidopsis thaliana
Source: PLoS One. 2010 Dec 8;5(12):e14255. doi: 10.1371/journal.pone.0014255 (PMC2999534; doi:10.1371/journal.pone.0014255)
Supplement: Table S1 — Significant differential phosphorylated peptides after MeJA, SA or SA/MeJA treatment compared to control. (0.48 MB DOC) [file pone.0014255.s002.doc]

**Table S1**

**Significant differential phosphorylated peptides after MeJA, SA or SA/MeJA treatment compared to control.**

| **Spot #** | **p-value** | **Treatment** | **P** | **Sequence** | **P-site** | **Kinase** | **Target protein** |
| --- | --- | --- | --- | --- | --- | --- | --- |
| 22 | 0.008 | SAJA | ▲ | QLSTSEENS | S-146 | CKI | SWISS;P02663;CAS2_BOVIN |
| 32 | 0.014 | JA | ▲ | YSGHSMSDP | S-289 | pyruvate dehydrogenase kinase | SWISS;P26267;ODPA_ASCSU |
| 38 | 0.046 | SAJA | ▲ | PRPASVPPS | S-59 | GSK3 | SWISS;P13834;UGS1_RABIT |
| 52 | 0.033 | SA | ▲ | EEGISQESS | S-87 |  | SWISS;P17095;HMGY_MOUSE |
| 53 | 0.040 | SAJA | ▼ | EQQQTEDEL | T-56 | CKII | SWISS;P02666;CASB_BOVIN |
| 60 | 0.023 | SA | ▼ | FPVSYSSSG | Y-687 |  | SWISS;P07949;RET_HUMAN |
| 78 | 0.000 | SA | ▼ | STSLSPFYL | S-45 | crystalline | SWISS;P02510;CRAB_BOVIN |
| 83 | 0.019 | SA | ▼ | STNDSLL | S-411 | GRK5 | SWISS;P07550;B2AR_HUMAN |
| 88 | 0.014 | JA | ▲ | STLASSFKR | S-889 | PKC | SWISS;Q05586;P35437;NMZ1_HUMAN |
| 99 | 0.049 | SAJA | ▼ | ILDTTGQEE | T-59 | transforming | SWISS;P01117;RASK_MSVKI |
| 100 | 0.021 | SA | ▼ | NDSNYIVKG | Y-809 | auto-P | SWISS;P07333;KFMS_HUMAN |
| 115 | 0.046 | SAJA | ▲ | NDMTSL | S-283 | tropomyosin | SWISS;P02558;TPMA_RABIT |
| 116 | 0.038 | SAJA | ▲ | AARLSLTDP | S-191 | PKA | SWISS;P13224;GPBB_HUMAN |
| 120 | 0.048 | JA | ▼ | RGRASSHSS | S-403 | PKC | SWISS;P02545;LAMA_HUMAN |
| 133 | 0.002 | SAJA | ▲ | WLTKSPDGN | S-491 | p34cdc2 | SWISS;Q62736;CALD_RAT |
| 136 | 0.015 | SAJA | ▲ | QEPGSGPPE | S-351 |  | SWISS;P30936;SSR3_RAT |
| 136 | 0.026 | JA | ▲ | QEPGSGPPE | S-351 |  | SWISS;P30936;SSR3_RAT |
| 150 | 0.017 | JA | ▲ | EIVESLSSS | S-30 | CK | SWISS;P02666;CASB_BOVIN |
| 153 | 0.050 | SAJA | ▲ | NRAITARRQ | T-11 | PhK | SWISS;P02643;TRIF_RABIT |
| 158 | 0.040 | SAJA | ▲ | GGRDSRSGS | S-160 | PKA | SWISS;P02687;MBP_BOVIN |
| 160 | 0.021 | SAJA | ▲ | EGVKSDQAE | S-536 |  | SWISS;P12839;NFM_RAT |
| 162 | 0.047 | SA | ▲ | RVRMSADAM | S-117 | PKA (PHK) | SWISS;P02643;TRIF_RABIT |
| 164 | 0.031 | SA | ▲ | SSKRAK | S-2 | PKC | SWISS;P02612;MLRM_CHICK |
| 166 | 0.037 | JA | ▲ | AVDRYIAIT | Y-132 | INSR | SWISS;P04274;B2AR_MESAU |
| 167 | 0.023 | JA | ▲ | DPLLTYRFP | T-480 | PKC | SWISS;P02545;LAMA_HUMAN |
| 168 | 0.006 | SA | ▲ | GENIYIRHS | Y-627 | EGFR | SWISS;P11171;41_HUMAN |
| 168 | 0.040 | SAJA | ▲ | GENIYIRHS | Y-627 | EGFR | SWISS;P11171;41_HUMAN |
| 169 | 0.021 | JA | ▲ | RLSPSPTSQ | S-392 | cdc2 | SWISS;P11516;LAMC_MOUSE |
| 169 | 0.026 | SAJA | ▲ | RLSPSPTSQ | S-392 | cdc2 | SWISS;P11516;LAMC_MOUSE |
| 171 | 0.024 | SAJA | ▲ | PLSRTLS | S-3 | CKI | SWISS;P13834;UGS1_RABIT |
| 172 | 0.045 | SAJA | ▲ | RSRASTPPA | S-47 | GSK3 | SWISS;P00515;KAP2_BOVIN |
| 174 | 0.037 | SAJA | ▲ | LRRASLAG |  | PKA/AGC1&2 | BOS TAURUS (BOVINE) LIVER PYRUVATE KINASE |
| 176 | 0.011 | SAJA | ▲ | THERSPSPS | S-329 |  | SWISS;P19836;CTPT_RAT |
| 178 | 0.010 | SAJA | ▲ | YETDYYRKG | Y-1189 | INSR | SWISS;P06213;INSR_HUMAN |
| 179 | 0.016 | SAJA | ▲ | RKQISVRGL | S-15 |  | SWISS;P11217;PHS2_HUMAN |
| 179 | 0.017 | JA | ▲ | RKQISVRGL | S-15 |  | SWISS;P11217;PHS2_HUMAN |
| 181 | 0.006 | SAJA | ▲ | LRRASVA | S-43 | PKA/AGC1&2 | SWISS;P12928;P04763;Q64618;KPYR_RAT |
| 181 | 0.020 | JA | ▲ | LRRASVA | S-43 | PKA/AGC1&2 | SWISS;P12928;P04763;Q64618;KPYR_RAT |
| 182 | 0.025 | JA | ▲ | YRGYSLGNW | S-42 | PKA | SWISS;P00698;Q90884;LYC_CHICK |
| 182 | 0.040 | SA | ▲ | YRGYSLGNW | S-42 | PKA | SWISS;P00698;Q90884;LYC_CHICK |
| 185 | 0.026 | SAJA | ▲ | RSKRSGSV |  |  | SWISS;P12798;KPBB_RABIT |
| 185 | 0.033 | SA | ▲ | RSKRSGSV |  |  | SWISS;P12798;KPBB_RABIT |
| 186 | 0.044 | SAJA | ▲ | TEGQYQQQP | Y-503 |  | SWISS;P42683;LCK_CHICK |
| 189 | 0.000 | SAJA | ▲ | VKRGISGL | S-48 |  | SWISS;P02304;P02305;H4_HUMAN |
| 189 | 0.003 | JA | ▲ | VKRGISGL | S-48 |  | SWISS;P02304;P02305;H4_HUMAN |
| 192 | 0.003 | SAJA | ▲ | QRRHSLEPP | S-17 | PKA | SWISS;P00526;SRC_RSVP |
| 193 | 0.001 | SA | ▲ | SRTLSVSSL | S-7 | AMP-PK SnRK | SWISS;P13834;UGS1_RABIT |
| 195 | 0.019 | SAJA | ▲ | LRGRSFMNN | S-376 | PKA | SWISS;P00511;K6PF_RABIT |
| 195 | 0.036 | JA | ▲ | LRGRSFMNN | S-376 | PKA | SWISS;P00511;K6PF_RABIT |
| 199 | 0.031 | JA | ▼ | EDNEYTARP | Y-413 | Src auto-P | SWISS;P31693;SRC_RSVPA |
| 209 | 0.002 | SAJA | ▼ | DYDSSDIED | S-468 | CKII | SWISS;Q03017;CACT_DROME |
| 216 | 0.009 | SA | ▼ | DDEMTGYVA | T-180 | MAPKK | SWISS;P47811;MP38_MOUSE |
| 216 | 0.021 | SAJA | ▼ | DDEMTGYVA | T-180 | MAPKK | SWISS;P47811;MP38_MOUSE |
| 217 | 0.022 | SA | ▼ | ETDYYRKGG | Y-1190 | INSR, auto-P | SWISS;P06213;INSR_HUMAN |
| 220 | 0.029 | SAJA | ▼ | RRAASVA |  | PKA/AGC1&2 | SWISS;P12928;P04763;Q64618;KPYR_RAT |
| 220 | 0.034 | JA | ▼ | RRAASVA |  | PKA/AGC1&2 | SWISS;P12928;P04763;Q64618;KPYR_RAT |
| 238 | 0.013 | SAJA | ▼ | PSLPTPPTR | T-208 | PDK1 (PDPK) | SWISS;P19332;TAU_RAT |
| 241 | 0.041 | SA | ▼ | PAPAVRASDRA |  | PHK | SWISS;P02646;TRIC_RABIT |
| 251 | 0.004 | SA | ▲ | MLDHSESTK | S-226 |  | SWISS;P27573;MYP0_MOUSE |
| 255 | 0.025 | SA | ▲ | DIPESQMEE | S-29 | CK | SWISS;P02730;B3AT_HUMAN |
| 256 | 0.025 | SAJA | ▲ | FPRASFGSR | S-29 | PKA | SWISS;P02542;DESM_CHICK |
| 256 | 0.050 | JA | ▲ | FPRASFGSR | S-29 | PKA | SWISS;P02542;DESM_CHICK |
| 269 | 0.009 | JA | ▲ | VRRISGL | not present |  | SWISS;P02304;P02305;H4_HUMAN |
| 271 | 0.027 | JA | ▲ | SETKTEEEE | T-1258 | CKII | SWISS;P06786;TOP2_YEAST |
| 275 | 0.036 | SAJA | ▼ | TRAPSRTAS | S-450 | MFPK | SWISS;P16638;ACLY_RAT |
| 298 | 0.025 | SA | ▲ | ERSQSRKDS | S-400 | PKC | SWISS;P15823;A1AB_RAT |
| 298 | 0.034 | SAJA | ▲ | ERSQSRKDS | S-400 | PKC | SWISS;P15823;A1AB_RAT |
| 320 | 0.037 | JA | ▼ | SIADTFVGT | T-363 | PDK1 (autoP) | SWISS;P06784;STE7_YEAST |
| 335 | 0.017 | SA | ▲ | PKDPSQRRR | S-11 | PKC | SWISS;P00523;Q91345;Q92013;SRC_CHICK |
| 335 | 0.030 | SAJA | ▲ | PKDPSQRRR | S-11 | PKC | SWISS;P00523;Q91345;Q92013;SRC_CHICK |
| 339 | 0.030 | SAJA | ▲ | QAGMTAPGT | T-220 |  | SWISS;P26932;CLPO_CHICK |
| 340 | 0.039 | SAJA | ▼ | SRRASRPVR | S-10 | PKA | SWISS;P02336;PRT2_CLUPA |
| 343 | 0.039 | SA | ▼ | QLNDSSEEE | S-31 | CKII | SWISS;P03129;VE7_HPV16 |
| 347 | 0.024 | SAJA | ▲ | ESRISLPLP | S-411 | auto-P | SWISS;P20152;VIME_MOUSE |
| 347 | 0.046 | SA | ▲ | ESRISLPLP | S-411 | auto-P | SWISS;P20152;VIME_MOUSE |
| 349 | 0.014 | SAJA | ▲ | NPGFYVEAN | Y-783 | EGFR | SWISS;P08487;PIP4_BOVIN |
| 354 | 0.008 | SA | ▼ | GGRASDYKS | S-131 | PKA | SWISS;P02687;MBP_BOVIN |
| 356 | 0.021 | JA | ▲ | EGTHSTKRG | S-618 | PKC | SWISS;P02671;FIBA_HUMAN |
| 356 | 0.027 | SAJA | ▲ | EGTHSTKRG | S-618 | PKC | SWISS;P02671;FIBA_HUMAN |
| 357 | 0.003 | SAJA | ▲ | ESLESYEIN | S-28 |  | SWISS;P07507;MGP_BOVIN |
| 362 | 0.034 | JA | ▲ | AVDGYVKPQ | Y-694 | JAK, TyrK,  no S/T | SWISS;P42229;STA5_HUMAN |
| 364 | 0.020 | SAJA | ▲ | GEINTEDDD | T-381 | CKII | SWISS;P31235;CAQS_RABIT |
| 367 | 0.047 | JA | ▲ | PGSPQKR | S-3 | sperm-specific | SWISS;P02256;H1_PARAN |
| 371 | 0.024 | JA | ▲ | ADGVYAASG | Y-368 | v-Fes, autoP | SWISS;P00543;FES_FSVST |
| 371 | 0.032 | SAJA | ▲ | ADGVYAASG | Y-368 | v-Fes, autoP | SWISS;P00543;FES_FSVST |
| 374 | 0.019 | SAJA | ▲ | YDKEYYSVH | Y-1234 | auto-P | SWISS;P08581;MET_HUMAN |
| 374 | 0.045 | SA | ▲ | YDKEYYSVH | Y-1234 | auto-P | SWISS;P08581;MET_HUMAN |
| 375 | 0.031 | SAJA | ▲ | RKLKSQGTR | S-8 |  | SWISS;P22613;STP1_SHEEP |
| 377 | 0.030 | SAJA | ▲ | LRRASPG |  | PKA/AGC1&2 | BOS TAURUS (BOVINE) LIVER PYRUVATE KINASE |
| 377 | 0.038 | SA | ▲ | LRRASPG |  | PKA/AGC1&2 | BOS TAURUS (BOVINE) LIVER PYRUVATE KINASE |
| 378 | 0.008 | SAJA | ▲ | YMAPYDNYV | Y-775 |  | SWISS;P09619;PGDR_HUMAN |
| 389 | 0.047 | SA | ▲ | SRTASFSES | S-454 | PKA, AKT | SWISS;P16638;ACLY_RAT |
| 391 | 0.047 | SAJA | ▲ | LRGPSWDPF | S-15 | MAPKAPK2 | SWISS;P04792;HS27_HUMAN |
| 406 | 0.042 | JA | ▼ | EKHHSIDAQ | S-15 | PEPCk | SWISS;P04711;CAP1_MAIZE |
| 422 | 0.023 | JA | ▲ | TKSASFLKG | S-262 | CDPK | SWISS;P08995;NO26_SOYBN |
| 443 | 0.021 | JA | ▼ | LVMQTAAGT | T-166 |  | SWISS;P25323;KMLC_DICDI |
| 451 | 0.030 | SA | ▼ | DHSRSTKAA | S-228 |  | SWISS;P27573;MYP0_MOUSE |
| 451 | 0.035 | JA | ▼ | DHSRSTKAA | S-228 |  | SWISS;P27573;MYP0_MOUSE |
| 454 | 0.041 | SA | ▼ | EDVGSDEED | S-254 | CKII | SWISS;P08238;HS9B_HUMAN |
| 458 | 0.020 | SA | ▼ | PGTESFVNA | S-391 | beta-ARK SnRK | SWISS;P04274;B2AR_MESAU |
| 458 | 0.037 | JA | ▼ | PGTESFVNA | S-391 | beta-ARK SnRK | SWISS;P04274;B2AR_MESAU |
| 476 | 0.048 | JA | ▲ | VRLRSSVPG | S-72/73 | auto-P | SWISS;P20152;VIME_MOUSE |
| 496 | 0.018 | SA | ▲ | NEEESSYSY | S-116  S-117  S-119 |  | SWISS;P20338;RRPP_HRSVL; P14156 |
| 499 | 0.016 | SA | ▼ | ASATSSSGG |  | CKI | SWISS;P13834;UGS1_RABIT |
| 507 | 0.036 | JA | ▼ | MSVEEV | S-2 | CKII | SWISS;P07260;IF4E_YEAST |
| 511 | 0.002 | SAJA | ▲ | FRRLSISTE | S-1018 | AMP-PK | SWISS;P18688;KPB1_RABIT |
| 518 | 0.012 | SA | ▲ | RRLSSLRA | S-235  S-236 | S6K | SWISS;P10660;P08227;RS6_HUMAN |
| 519 | 0.021 | JA | ▼ | TAESSQAEE | S-206/7 | CKII | SWISS;P06836;NEUM_BOVIN |
| 525 | 0.033 | SAJA | ▲ | VVGGSLRGA | S-378 | PKC | SWISS;P18031;PTN1_HUMAN |
| 526 | 0.017 | SA | ▲ | SRRPSYRKI | S-133 | PKA | SWISS;P16220;P21934;CREB_HUMAN |
| 526 | 0.045 | JA | ▼ | SRRPSYRKI | S-133 | PKA | SWISS;P16220;P21934;CREB_HUMAN |
| 534 | 0.002 | SAJA | ▼ | LDDQYTSSS | Y-518 |  | SWISS;P24604;TEC_MOUSE |
| 548 | 0.004 | SAJA | ▲ | DADEYLIPQ | Y-1016 | EGFR | SWISS;P00533;P06268;EGFR_HUMAN |
| 550 | 0.026 | SA | ▼ | GGLTSPGLS | S-431 | MAPK | SWISS;P05787;K2C8_HUMAN |
| 551 | 0.033 | SAJA | ▲ | GTVPSDNID | S-396 | GRK2 | SWISS;P07550;B2AR_HUMAN |
| 559 | 0.005 | SAJA | ▲ | DPGTSYRTR | S-296 | pyruvate dehydrogenase kinase | SWISS;P26267;ODPA_ASCSU |
| 559 | 0.049 | JA | ▲ | DPGTSYRTR | S-296 | pyruvate dehydrogenase kinase | SWISS;P26267;ODPA_ASCSU |
| 560 | 0.002 | JA | ▲ | GEGTYGVVY | Y-15 | WEE/MYT | SWISS;P13863;CC2_CHICK |
| 561 | 0.046 | SAJA | ▲ | RLRLSPSPT | S-390 | cdc2 | SWISS;P11516;LAMC_MOUSE |
| 567 | 0.028 | SA | ▲ | ADGIYAASG | Y-500 | v-Fes | SWISS;P00542;FES_FSVGA |
| 576 | 0.028 | JA | ▲ | PLSRTLSVS | S-7 | PKC/CAMII | glycogen (starch) synthase, muscle |
| 585 | 0.045 | SAJA | ▲ | SRSRTPSLP | T-212 | GSK3 /PDK1 | PIR;2144820 |
| 609 | 0.003 | SA | ▼ | ETAESSQAE | S-206/7 | CKII | SWISS;P06836;NEUM_BOVIN |
| 609 | 0.047 | JA | ▼ | ETAESSQAE | S-206/7 | CKII | SWISS;P06836;NEUM_BOVIN |
| 610 | 0.027 | JA | ▲ | YTRFSLARQ | S-24 | PKC | SWISS;P02786;TRSR_HUMAN |
| 633 | 0.018 | JA | ▼ | PLSRTLSVRSL |  |  | SWISS;P13834;UGS1_RABIT |
| 636 | 0.049 | SAJA | ▼ | KRRRSSKDT | S-896 | PKC | SWISS;Q05586;P35437;NMZ1_HUMAN |
| 646 | 0.010 | SA | ▼ | APVASPAAP | S-62 | MAPK | SWISS;P17599;SYN1_BOVIN |
| 650 | 0.009 | SAJA | ▼ | EDTLSDSDD | S-261 | CKII | SWISS;P04198;MYCN_HUMAN |
| 653 | 0.004 | JA | ▼ | SFMDSSGLG | S-58 | spoIIAB | SWISS;P10727;SP21_BACSU |
| 654 | 0.041 | SAJA | ▼ | QKRPSQRSK | S-7 | PKA | SWISS;P02687;MBP_BOVIN |
| 659 | 0.019 | SAJA | ▼ | RDPVTENAV | T-271 | GRK, G-Prot coupled receptor K | SWISS;P08172;ACM2_HUMAN |
| 668 | 0.040 | SA | ▼ | VNATYVNVK | Y-1356 |  | SWISS;P08581;MET_HUMAN |
| 672 | 0.014 | SAJA | ▲ | VRKRTLRRL | T-678 | PKC | SWISS;P00533;P06268;EGFR_HUMAN |
| 675 | 0.004 | SA | ▲ | PSQRSKYLA | S-10 | PKA | SWISS;P02687;MBP_BOVIN |
| 681 | 0.009 | JA | ▼ | KSKISASRK | S-43 | PKC | SWISS;P08057;TRIC_BOVIN |
| 681 | 0.011 | SA | ▼ | KSKISASRK | S-43 | PKC | SWISS;P08057;TRIC_BOVIN |
| 682 | 0.014 | SA | ▼ | KRKQISVGGL | S-15 | PHK (phosphorylase kinase) | SWISS;P11217;PHS2_HUMAN |
| 690 | 0.022 | SAJA | ▲ | ERRVSNAGG | S-157 | PKA, PKC, PKG, ROCKI | SWISS;P50552;VASP_HUMAN |
| 691 | 0.011 | SAJA | ▲ | IREESPPHS | S-343 |  | SWISS;P09258;VGLI_VZVD |
| 692 | 0.039 | JA | ▲ | NDSVYANWM | Y-1096 |  | SWISS;P07949;RET_HUMAN |
| 698 | 0.019 | SA | ▼ | GSDVSFNEE | S-1423 | CKII | SWISS;P06786;TOP2_YEAST |
| 700 | 0.012 | SAJA | ▲ | ERQKTQTKL | T-289 | MLCK | SWISS;P25323;KMLC_DICDI |
| 708 | 0.006 | SAJA | ▲ | RFTDTRKDE | T-59 | CaM-III | SWISS;P13639;EF2_HUMAN |
| 714 | 0.037 | SAJA | ▲ | TIAVG | T-1 | STN8 | SWISS;P06005;PSBD_SPIOL |
| 714 | 0.043 | JA | ▲ | TIAVG | T-1 | STN8 | SWISS;P06005;PSBD_SPIOL |
| 725 | 0.029 | SAJA | ▲ | STTVSKTET | S-338 |  | SWISS;P02700;OPSD_SHEEP |
| 726 | 0.050 | JA | ▲ | TRKISASEF | S-92 | PKA? PKG | GENBANK;L16545 |
| 727 | 0.021 | JA | ▲ | PGPQSPGSP | S-345 | MAPK ? | SWISS;P14598;NCF1_HUMAN |
| 735 | 0.005 | SAJA | ▲ | QKAQTERKS | T-190 | PKC | SWISS;P13789;TRT1_BOVIN |
| 735 | 0.038 | JA | ▲ | QKAQTERKS | T-190 | PKC | SWISS;P13789;TRT1_BOVIN |
| 743 | 0.039 | SAJA | ▲ | GSPGTPGSR | T-205 | p34cdc2-p58cyclin / PDK1 | PIR;2144820 |
| 746 | 0.012 | SAJA | ▲ | GGIRSLNVA | S-113 | isocitrate | SWISS;P08200;IDH_ECOLI |
| 747 | 0.032 | SA | ▲ | GTRLSLARM | S-34 | PKA | SWISS;Q28115;GFAP_BOVIN |
| 747 | 0.043 | SAJA | ▲ | GTRLSLARM | S-34 | PKA | SWISS;Q28115;GFAP_BOVIN |
| 749 | 0.030 | SAJA | ▲ | ESHESMESY | S-25 |  | SWISS;P08493;MGP_HUMAN |
| 749 | 0.039 | SA | ▲ | ESHESMESY | S-25 |  | SWISS;P08493;MGP_HUMAN |
| 752 | 0.014 | SAJA | ▲ | RRPTPA | T-35 |  | SWISS;P01099;IPP1_RABIT |
| 752 | 0.015 | JA | ▲ | RRPTPA | T-35 |  | SWISS;P01099;IPP1_RABIT |
| 755 | 0.004 | SAJA | ▲ | DNPDYQQDF | Y-1172 | EGFR | SWISS;P00533;P06268;EGFR_HUMAN |
| 756 | 0.035 | JA | ▲ | GDVKYADIE | Y-763 | Tyr-K, no S/T | SWISS;P09619;PGDR_HUMAN |
| 762 | 0.031 | JA | ▲ | RRASVA | S-43 | PKA/AGC1&2 | SWISS;P12928;P04763;Q64618;KPYR_RAT |
| 763 | 0.036 | SAJA | ▲ | ADDEYAPKQ | Y-434 | c-Src | SWISS;P00528;SRC1_DROME |
| 764 | 0.001 | SAJA | ▲ | TESQYQQQP | Y-522 |  | SWISS;P08631;HCK_HUMAN |
| 766 | 0.020 | SAJA | ▲ | WTSDSAGEE | S-232 | CKI | SWISS;P27348;143T_HUMAN |
| 769 | 0.036 | SAJA | ▲ | LRRASGG |  | PKA/AGC1&2 | BOS TAURUS (BOVINE) LIVER PYRUVATE KINASE |
| 772 | 0.005 | SA | ▲ | PLAGSPVIA | S-370 | cdc2 | SWISS;P19138;P20426;KC21_HUMAN |
| 774 | 0.010 | SAJA | ▲ | TEDQYSLVE | Y-607 | c-Src | SWISS;P27986;P85A_HUMAN |
| 774 | 0.029 | JA | ▲ | TEDQYSLVE | Y-607 | c-Src | SWISS;P27986;P85A_HUMAN |
| 777 | 0.012 | SAJA | ▲ | RTKRSGSV | S-26 | PKC | phosphorylase b kinase beta regulatory chain |
| 778 | 0.011 | SAJA | ▲ | KREASLDNQ | S-598 | PKA | SWISS;P06593;PHY3_AVESA |
| 778 | 0.024 | JA | ▲ | KREASLDNQ | S-598 | PKA | SWISS;P06593;PHY3_AVESA |
| 780 | 0.013 | JA | ▲ | QQGMTVYGL | T-259 |  | SWISS;P26932;CLPO_CHICK |
| 784 | 0.001 | SAJA | ▲ | QRRTSLTGS | S-1757 | PKA | SWISS;P07293;CIC1_RABIT |
| 804 | 0.031 | SAJA | ▼ | DDAYSDTET | S-86 | CKI | SWISS;P11845;IPP2_RABIT |
| 804 | 0.045 | JA | ▼ | DDAYSDTET | S-86 | CKI | SWISS;P11845;IPP2_RABIT |
| 812 | 0.009 | SA | ▼ | KEAKSD | S-99 |  | SWISS;P02316;HG14_BOVIN |
| 812 | 0.022 | JA | ▼ | KEAKSD | S-99 |  | SWISS;P02316;HG14_BOVIN |
| 826 | 0.000 | SA | ▼ | PRRNSRASL | S-573 | PKA | SWISS;P04775;CIN2_RAT |
| 837 | 0.044 | SAJA | ▼ | EQESSGEED | S-121 | CKII | SWISS;P11845;IPP2_RABIT |
| 839 | 0.021 | JA | ▼ | MGEASGAQL | S-369 | beta-ARK SnRK | SWISS;P04274;B2AR_MESAU |
| 855 | 0.014 | JA | ▼ | RDEEYGYEA | Y-411 | Tyr-K, no S/T | SWISS;P21548;GAC2_CHICK |
| 857 | 0.032 | SA | ▼ | TKRSGSV | S-26 |  | SWISS;P12798;KPBB_RABIT |
| 861 | 0.002 | SA | ▼ | RRASS |  | PKA/AGC1&2 | SWISS;P12928;P04763;Q64618;KPYR_RAT |
| 862 | 0.039 | SAJA | ▼ | STPLSPTRI | S-22 | cdc2 | SWISS;P02545;LAMA_HUMAN |
| 869 | 0.007 | SAJA | ▼ | SRKESYSVY | S-36 | PKA | SWISS;P02278;H2B_HUMAN |
| 871 | 0.042 | SAJA | ▼ | PSPSSRVTV | S-395 | PKC | SWISS;P20700;LAM1_HUMAN |
| 881 | 0.022 | JA | ▼ | KTETSQVAP | S-343 | RhK | SWISS;P02699;OPSD_BOVIN |
| 883 | 0.015 | SAJA | ▼ | IGRFSEPHA | S-139 |  | SWISS;P00517;KAPA_BOVIN |
| 890 | 0.028 | JA | ▼ | GRPITPPRN | T-320 | cdc2 | SWISS;P08129;P22802;P20653;PP1A_HUMAN |
| 894 | 0.007 | JA | ▲ | GRVLTLPRS | T-1376 | PKC | SWISS;P15127;P97681;INSR_RAT |
| 909 | 0.006 | SA | ▲ | KSRRTI | T-271 | PKC | SWISS;P01589;IL2A_HUMAN |
| 928 | 0.047 | SAJA | ▲ | SRLHSVRER | S-15 |  | SWISS;P49036;SUS2_MAIZE |
| 930 | 0.048 | SA | ▲ | LGGGTFDIS | T-198 | DnaK, auto-P | SWISS;P04475;DNAK_ECOLI |
| 933 | 0.003 | SAJA | ▲ | NGDASPAAA | S-45 |  | SWISS;P12624;MACS_BOVIN |
| 933 | 0.041 | JA | ▲ | NGDASPAAA | S-45 |  | SWISS;P12624;MACS_BOVIN |
| 934 | 0.017 | SAJA | ▲ | EHVSSSEES | S-24 | CKI | SWISS;P02663;CAS2_BOVIN |
| 935 | 0.037 | SAJA | ▲ | ESMESYEVS | S-28 |  | SWISS;P08494;MGP_RAT |
| 937 | 0.016 | SAJA | ▲ | NMPSSDDGL | S-251 | GRK | SWISS;P08172;ACM2_HUMAN |
| 937 | 0.019 | JA | ▲ | NMPSSDDGL | S-251 | GRK | SWISS;P08172;ACM2_HUMAN |
| 940 | 0.027 | SAJA | ▲ | AVRRSDRAY | S-20 | PKA | SWISS;P02646;TRIC_RABIT |
| 943 | 0.016 | JA | ▲ | GSTSTPAPS | T-446 | MFPK (GSK3-like) | SWISS;P16638;ACLY_RAT |
| 945 | 0.036 | SA | ▲ | ESHESLESY | S-25 |  | SWISS;P07507;MGP_BOVIN |
| 948 | 0.002 | SAJA | ▲ | RRPSPA |  |  | SWISS;P12928;P04763;Q64618;KPYR_RAT |
| 951 | 0.001 | SAJA | ▲ | DNLYYWDQD | Y-1222 |  | SWISS;P04626;ERB2_HUMAN |
| 952 | 0.009 | SAJA | ▲ | GDSSYKNIH | Y-697 | c-Fms (autoP) | SWISS;P09581;KFMS_MOUSE |
| 952 | 0.020 | JA | ▲ | GDSSYKNIH | Y-697 | c-Fms (autoP) | SWISS;P09581;KFMS_MOUSE |
| 954 | 0.028 | SAJA | ▲ | ASGSFKL | S-103 | PKC | SWISS;P02253;H11_BOVIN |
| 955 | 0.019 | SAJA | ▲ | LRRGSLG |  | PKA/AGC1&2 | BOS TAURUS (BOVINE) LIVER PYRUVATE KINASE |
| 955 | 0.037 | JA | ▲ | LRRGSLG |  | PKA/AGC1&2 | BOS TAURUS (BOVINE) LIVER PYRUVATE KINASE |
| 961 | 0.025 | SA | ▲ | TTRVTPLRT | T-64 | cdc2 | SWISS;P02542;DESM_CHICK |
| 963 | 0.022 | SAJA | ▲ | RHRDTGILD | T-33 | PKA | SWISS;P02687;MBP_BOVIN |
| 964 | 0.030 | SAJA | ▲ | NRIYTHQVV | T-176 |  | SWISS;P20911;CDK7_XENLA |
| 967 | 0.031 | SAJA | ▲ | SSEESIISQ | S-28 | CKI | SWISS;P02663;CAS2_BOVIN |
| 973 | 0.045 | SAJA | ▲ | RTKGSGSV |  |  | SWISS;P12798;KPBB_RABIT |
| 977 | 0.021 | SA | ▲ | SRSRSRSPG | S-82 | RS | SWISS;P23913;LBR_CHICK |
| 978 | 0.031 | SAJA | ▲ | KRKVSSAEG | S-6 |  | SWISS;P02316;HG14_BOVIN |
| 979 | 0.025 | SAJA | ▲ | LQDDYEDMM | Y-8 |  | SWISS;P02730;B3AT_HUMAN |
| 985 | 0.046 | SAJA | ▼ | HHHATPSPP | T-355 | GSK3 | SWISS;P01103;MYB_CHICK |
| 1024 | 0.048 | JA | ▼ | KRPSFRAKA |  | AGC | SWISS;P02687;MBP_BOVIN |
| 1031 | 0.013 | SA | ▼ | LNRMSFASN | S-1200 | AMP-PK SnRK | SWISS;P11497;P97902;COAC_RAT |
| 1038 | 0.011 | SAJA | ▼ | APRTPGGRR |  | MAPK | SWISS;P02687;MBP_BOVIN |
| 1038 | 0.035 | SA | ▼ | APRTPGGRR |  | MAPK | SWISS;P02687;MBP_BOVIN |
| 1053 | 0.014 | SAJA | ▼ | STNDSPL | S-416 | beta-ARK | SWISS;P04274;B2AR_MESAU |
| 1059 | 0.005 | SAJA | ▼ | TPPLSPIDM | S-243 | ERT | SWISS;P05412;AP1_HUMAN |
| 1060 | 0.007 | SA | ▼ | VKGATSDEE | T-1086 | CKII | SWISS;P06786;TOP2_YEAST |
| 1060 | 0.013 | SAJA | ▼ | VKGATSDEE | T-1086 | CKII | SWISS;P06786;TOP2_YEAST |
| 1073 | 0.031 | SAJA | ▼ | KRSNSVDTS | S-129 | PKA | SWISS;P13834;UGS1_RABIT |
| 1076 | 0.022 | SA | ▼ | KKDVTPVKA | T-53 | CDC2-like | SWISS;P10156;H1_TETTH |
| 1077 | 0.011 | JA | ▼ | KSRWSGSQQ | S-499 | PKC | SWISS;P04049;KRAF_HUMAN |
| 1077 | 0.015 | SAJA | ▼ | KSRWSGSQQ | S-499 | PKC | SWISS;P04049;KRAF_HUMAN |
| 1130 | 0.004 | SAJA | ▲ | EHIPYTHMN | Y-1361 | INSR (autoP) | SWISS;P06213;INSR_HUMAN |
| 1130 | 0.007 | JA | ▲ | EHIPYTHMN | Y-1361 | INSR (autoP) | SWISS;P06213;INSR_HUMAN |
| 1131 | 0.045 | JA | ▲ | ESMESYELN | S-28 |  | SWISS;P08493;MGP_HUMAN |
| 1138 | 0.042 | SAJA | ▲ | GFKRSYEEH | S-1354 |  | SWISS;P06213;INSR_HUMAN |
| 1139 | 0.003 | SA | ▲ | GSRRRRRRY |  |  | SWISS;P15340;HSP_CHICK |
| 1139 | 0.012 | SAJA | ▲ | GSRRRRRRY |  |  | SWISS;P15340;HSP_CHICK |
| 1140 | 0.020 | SA | ▲ | EFPLSPPKK | S-37 | cdc2 | SWISS;P16949;STHM_HUMAN |
| 1148 | 0.024 | SAJA | ▲ | GDSESGEEE | S-85 |  | SWISS;P11831;SRF_HUMAN |
| 1149 | 0.003 | SAJA | ▲ | RKRKSSQAL | S-28 | PKA | SWISS;P04625;THA_CHICK |
| 1151 | 0.038 | SA | ▲ | LRRATLG |  | PKA/AGC1&2 | BOS TAURUS (BOVINE) LIVER PYRUVATE KINASE |
| 1151 | 0.044 | JA | ▲ | LRRATLG |  | PKA/AGC1&2 | BOS TAURUS (BOVINE) LIVER PYRUVATE KINASE |
| 1152 | 0.017 | JA | ▲ | RRSSSVGYI | S-383 | PKA | SWISS;P02718;ACHD_TORCA |
| 1152 | 0.018 | SAJA | ▲ | RRSSSVGYI | S-383 | PKA | SWISS;P02718;ACHD_TORCA |
| 1153 | 0.011 | SAJA | ▲ | SKVTSKAGS | S-320 |  | PIR;2144820 |
| 1153 | 0.031 | SA | ▲ | SKVTSKAGS | S-320 |  | PIR;2144820 |
| 1154 | 0.040 | JA | ▲ | RGYSLG | S-42 | PKA | SWISS;P00698;Q90884;LYC_CHICK |
| 1156 | 0.002 | SA | ▲ | TEPQYQPGE | Y-530 |  | SWISS;P06241;FYN_HUMAN |
| 1159 | 0.025 | SA | ▲ | RGRSSVYSA | S-311 | myosin | SWISS;P10569;MYSC_ACACA |
| 1159 | 0.031 | JA | ▲ | RGRSSVYSA | S-311 | myosin | SWISS;P10569;MYSC_ACACA |
| 1159 | 0.039 | SAJA | ▲ | RGRSSVYSA | S-311 | myosin | SWISS;P10569;MYSC_ACACA |
| 1160 | 0.041 | SAJA | ▲ | RKRTLRRL | T-678 |  | SWISS;P00533;P06268;EGFR_HUMAN |
| 1162 | 0.025 | JA | ▲ | YGNGYSSNS | Y-354 | INSR | SWISS;P04274;B2AR_MESAU |
| 1163 | 0.026 | SA | ▲ | SRTPSLPTP | S-214 | PKA | PIR;2144820 |
| 1167 | 0.036 | JA | ▲ | TRRLTGFLP | T-30 |  | SWISS;P06738;PHSG_YEAST |
| 1172 | 0.032 | SAJA | ▲ | QMALTPVVV | T-172 | CAK | SWISS;P30285;CDK4_MOUSE |
| 1173 | 0.045 | JA | ▲ | SRSRSPGRP | S-84 | RS | SWISS;P23913;LBR_CHICK |
| 1176 | 0.032 | JA | ▲ | QRRRSLEPP | S-16 | PKA | SWISS;P00523;Q91345;Q92013;SRC_CHICK |

***Footnote:*** The direction of phosphorylation (P) of substrates relative to control (C) compared to treatments with MeJA, SA or SA/MeJA is indicated with arrows (increased; ▲; green symbols or decreased;▼; red symbols). Spot number (Spot #), p-value, peptide sequence (phosphorylation site underlined), phosphorylation site (P-Site; amino acids numbered as present in the target protein), predicted kinase to phosphorylate the target petide, and target protein from which the peptide sequence is derived (SWISS annotation) are indicated and follow PepScan documentation (www.pepscanpresto.com). Plant specific substrates are shaded in yellow.
