# Supplementary material for: Kinome Profiling Reveals an Interaction Between Jasmonate, Salicylate and Light Control of Hyponastic Petiole Growth in Arabidopsis thaliana
Source: PLoS One. 2010 Dec 8;5(12):e14255. doi: 10.1371/journal.pone.0014255 (PMC2999534; doi:10.1371/journal.pone.0014255)
Supplement: Table S2 — Gene representation of functional classes differentially expressed upon MeJA treatment in the ‘photosynthesis’ sub-bin calculated by MAPMAN. (0.04 MB DOC) [file pone.0014255.s003.doc]

**Table S2**

**Gene representation of functional classes differentially expressed upon MeJA treatment in the ‘photosynthesis’ sub-bin calculated by MapMan**.

| **bin** | **name** | **elements** | **p-value 1 h** | **p-value 3 h** | **p-value 6 h** |
| --- | --- | --- | --- | --- | --- |
| 1.1 | PS.lightreaction | 122 | **6.5E-03** | **2.0E-08** | **4.2E-04** |
| 1.1.1 | PS.lightreaction.photosystem II | 49 | **1.9E-02** | **6.3E-06** | **5.7E-04** |
| 1.1.2 | PS.lightreaction.photosystem I | 23 | 6.5E-01 | 2.3E-01 | 9.8E-02 |
| 1.1.3 | PS.lightreaction.cytochrome b6/f | 8 | 6.9E-01 | 8.5E-01 | 9.8E-01 |
| 1.1.4 | PS.lightreaction.ATP synthase | 12 | 7.8E-01 | 4.4E-01 | 9.4E-01 |
| 1.1.5 | PS.lightreaction.other electron carrier (ox/red) | 13 | 5.3E-01 | 6.8E-01 | 9.6E-01 |
| 1.1.6 | PS.lightreaction.NADH DH | 3 | 6.7E-01 | 8.2E-01 | 8.7E-01 |
| 1.2 | PS.photorespiration | 20 | 2.1E-01 | 1.0E-01 | 2.2E-01 |
| 1.3 | PS.calvin cycle | 35 | 1.2E-01 | **8.5E-03** | 2.4E-01 |

***Footnote:*** Bins show clusters of genes (elements) differentially expressed upon MeJA treatment. Significant probabilities (p<0.05) are designated in bold.
