# Supplementary material for: Kinome Profiling Reveals an Interaction Between Jasmonate, Salicylate and Light Control of Hyponastic Petiole Growth in Arabidopsis thaliana
Source: PLoS One. 2010 Dec 8;5(12):e14255. doi: 10.1371/journal.pone.0014255 (PMC2999534; doi:10.1371/journal.pone.0014255)
Supplement: Table S3 — Gene representation of functional classes differentially expressed upon MeJA treatment in the MAPMAN ‘hormone metabolism’ sub-bin. (0.05 MB DOC) [file pone.0014255.s004.doc]

**Table S3**

**Gene representation of functional classes differentially expressed upon MeJA treatment in the MapMan** **‘hormone metabolism’ sub-bin.**

| **bin** | **name** | **elements** | **p-value 1 h** | **p-value 3 h** | **p-value 6 h** |
| --- | --- | --- | --- | --- | --- |
| 17.1 | hormone metabolism.abscisic acid | 45 | 8.8E-01 | 9.8E-01 | 8.1E-01 |
| 17.2 | hormone metabolism.auxin | 208 | 2.1E-01 | **8.5E-03** | **2.8E-04** |
| 17.2.1 | hormone metabolism.auxin.synthesis-degradation | 23 | 6.0E-02 | **1.9E-02** | **1.5E-03** |
| 17.2.2 | hormone metabolism.auxin.signal transduction | 43 | 5.2E-01 | 4.2E-01 | 4.1E-01 |
| 17.2.3 | hormone metabolism.auxin.induced-regulated-responsive-activated | 142 | **3.3E-02** | **1.0E-04** | **3.1E-08** |
| 17.3 | hormone metabolism.brassinosteroid | 46 | 5.9E-01 | 6.5E-01 | 2.1E-01 |
| 17.4 | hormone metabolism.cytokinin | 49 | **4.3E-02** | **9.4E-03** | **2.9E-02** |
| 17.4.1 | hormone metabolism.cytokinin.synthesis-degradation | 18 | 9.5E-01 | 9.0E-01 | 7.7E-01 |
| 17.4.2 | hormone metabolism.cytokinin.signal transduction | 31 | **3.6E-03** | **9.2E-04** | **1.1E-02** |
| 17.5 | hormone metabolism.ethylene | 107 | 9.7E-01 | 2.7E-01 | 1.4E-01 |
| 17.6 | hormone metabolism.gibberelin | 49 | 6.3E-01 | 5.0E-01 | 2.8E-01 |
| 17.7 | hormone metabolism.jasmonate | 32 | 2.3E-01 | 5.0E-01 | 6.0E-01 |
| 17.8 | hormone metabolism.salicylic acid | 19 | 5.1E-01 | 3.8E-01 | 7.6E-01 |

***Footnote:*** The probability (p-values) that the genes within the bin are differentially expressed upon 1 h, 3 h or 6 h of MeJA treatment. Significant probabilities (p<0.05) are designated in bold.
