# Supplementary figures and images for: Kinome Profiling Reveals an Interaction Between Jasmonate, Salicylate and Light Control of Hyponastic Petiole Growth in Arabidopsis thaliana
Source: PLoS One. 2010 Dec 8;5(12):e14255. doi: 10.1371/journal.pone.0014255 (PMC2999534; doi:10.1371/journal.pone.0014255)

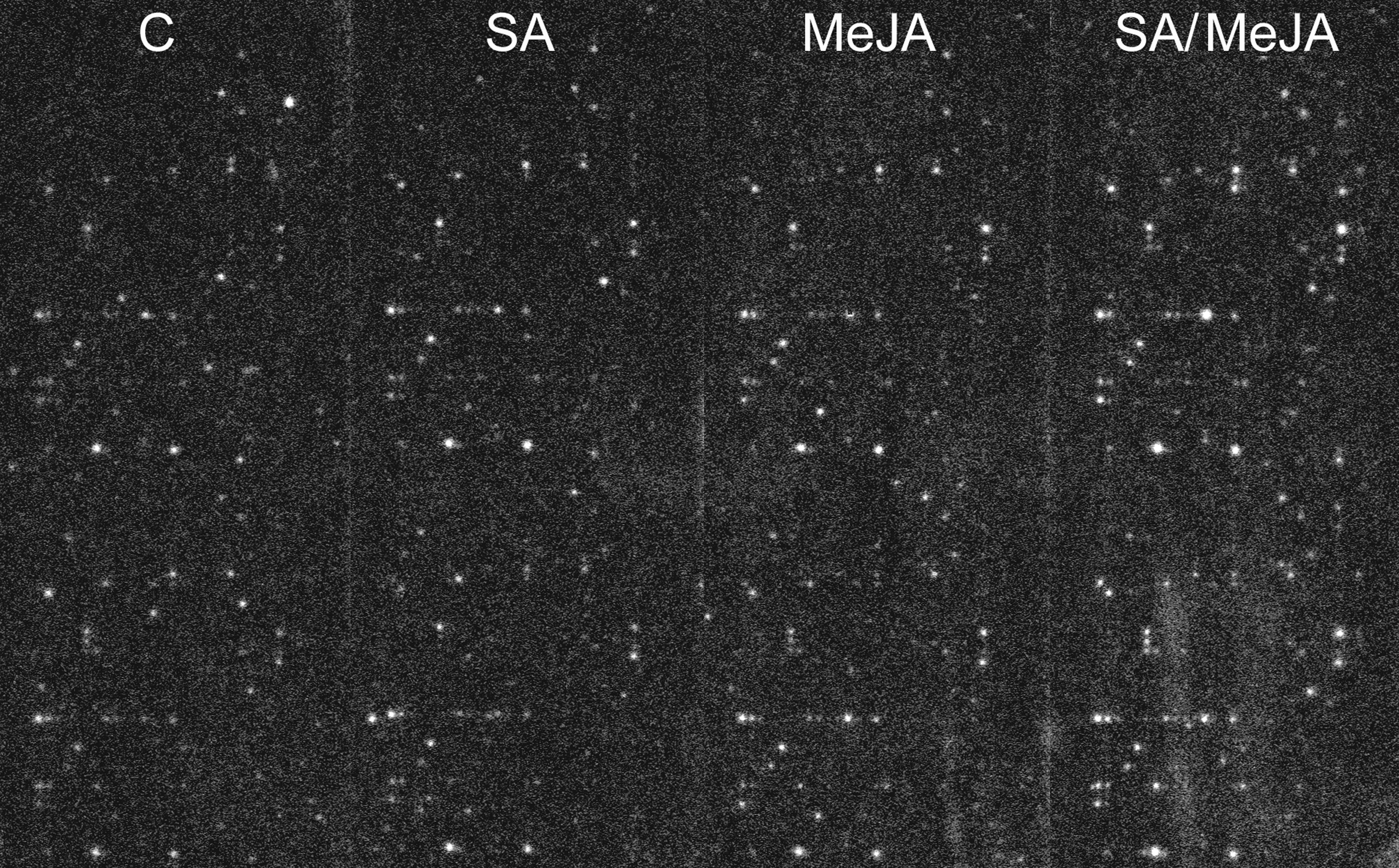

Supplement: Figure S1 — Typical PepChip autoradiogram. (10.43 MB TIF) [file pone.0014255.s005.tif]
