# Supplementary material for: Kinome Profiling Reveals an Interaction Between Jasmonate, Salicylate and Light Control of Hyponastic Petiole Growth in Arabidopsis thaliana
Source: PLoS One. 2010 Dec 8;5(12):e14255. doi: 10.1371/journal.pone.0014255 (PMC2999534; doi:10.1371/journal.pone.0014255)
Supplement: Text S1 — General analysis of observed kinome profiles. (0.05 MB DOC) [file pone.0014255.s006.doc]

**Text S1**

# General analysis of observed kinome profiles

In our PepChip analysis 37 substrate residues, annotated to detect Tyrosine (Tyr) phosphorylation were differentially, generally increased, phosphorylated (see Table S1). The artificial substrate Kemptide (RRASLG) is generally used to monitor Protein Kinase-A (PKA) activity. Several Kemptide analogues showed increased phosphorylation after our treatments (12 out of 15; Table S1). Two exceptions were the more divergent peptides RRAASVA and RRASS, which had decreased phosphorylation. Other PKA targets and also PKC (20 out of 27; Table S1) targets were generally increased. Substrates of the Casein Kinase II (CKII) showed generally decreased phosphorylation after treatment (13 out of 15).

Cell Division Cycle 2 (CDC2) is represented as a peptide substrate for the inhibitory kinases WEE and MYT (motif: GEGTYGVVY), which is identical in the Arabidopsis CDC2 orthologue (At3g48750). MeJA treatment increased phosphorylation, which suggests CDC2 inhibition. Generally, CDC2 target substrates were higher phosphorylated after SA and SA/MeJA treatment. One CDC2-annotated peptide was also higher after MeJA, but another showed lower MeJA-mediated phosphorylation. Two substrate peptides present in Pyruvate Dehydrogenase (PDH) had increased phosphorylation after MeJA treatment (one also after SA/MeJA) indicating activation of PDH-kinase. Both *Ascaris suum* (Pig roundworm) derived peptides are highly similar to Arabidopsis PDHs (At1g24180 and At1g59900).

Our PepChip analysis indicated differential phosphorylation of Tyr residues. The presence of receptor Tyr-kinases and soluble Tyr-kinases *in planta* are a matter of debate, however, accumulating evidence shows that Tyr-kinase activity is present in plants [1–3]. A specific example is WEE kinase that is known to direct Tyr-phosphorylation, but other (dual specificity) kinases with high Tyr-phosphorylation ability have been described [4–6]. Most of the identified Tyr peptides also have a Ser and/or Thr residue, so they can potentially be phosphorylated by Ser/Thr-kinases. In nine peptides no Ser or Thr is present, but eight of these have an Asp, which is phosphorylated in the response regulators of two component sensing systems, as known from e.g. cytokinin signaling [7,8]. None of theses Asp residues are present in a typical response regulator motif. One peptide (NPGFYVEAN) has only a Tyr as obvious phosphorylation residue. Therefore, we may have detected differences in Tyr-kinase activities; however the identity of these kinases is unknown.

PKA has not been identified *in planta*, however the AGC-kinases AGC1-1 and AGC1-2 were shown to phosphorylate the Kemptide peptide (RRASLG) and are therefore considered to be the plant PKA orthologues [9]. The two more divergent Kemptide analogues RRAASVA and RRASS (also mentioned above) might not have the right signature to be true Kemptide homologues and could therefore reflect phosphorylations by other (unknown) kinases. The PFC, another AGC-kinase, is also not *per se* found in plants. Both PKA and PKC substrates are generally phosphorylated to a higher extend, indicating that AGC kinases show higher activity after SA, MeJA, or SA/MeJA treatment.

CKII has a broad substrate consensus sequence and is implicated in for example development, cell cycle progression, chromatin remodeling, and circadian rhythm [10–13]. CKII substrates show generally lower phosphorylation after the treatments (Table S1). It remains unclear which specific processes are affected by this.

The CDC2 motif on the PepChip (derived from chicken CDC2) is identical to a sequence of the Arabidopsis CDC2 (CDKA, At3g48750). The Tyr15 residue is phosphorylated by WEE in Arabidopsis. WEE activity arrests the cell cycle i.e. inhibits CDC2 activity [14]. MeJA treatment differentially phosphorylated CDC2 substrates, indicating that MeJA might inhibit the cell cycle via WEE-mediated inhibitory phosphorylation of CDKA. This is in agreement with earlier observations that cell cycle progression is inhibited by JA [15,16].

Four plant specific proteins were differentially phosphorylated by MeJA treatment (Table 1). Aquaporins belong to the major intrinsic protein superfamily and are known for regulating water status during drought stress and processes such as petal opening and cell elongation [17–19]. The kinase that phosphorylates the specific aquaporin-derived sequence on the PepChip is from the large family of Calcium/Calmodulin derived protein kinase (CDPK) in Arabidopsis [20]. MeJA-directed enhanced phosphorylation of this indicates activation of the water channel [18,21]. However, this contrasts to the significant down regulation in transcription observed in bin 34.19; transport; major intrinsic proteins, i.e. the aquaporins, at 6 h (P = 3.5E-3).

Phosphoenol pyruvate carboxylase (PEP carboxylase) is phosphorylated by the highly specific PEP-carboxylase kinase in plants. PEP-carboxylase processes PEP during CO_2_ fixation in C4 and CAM plants. Although its function is less well described in C3 plants such as Arabidopsis, it acts particularly during germination and in providing the TCA/Krebs cycle with intermediates and is activated in response to nitrogen fixation e.g. water stress in C3 plants such as Arabidopsis [22,23]. The reduced phosphorylation observed after MeJA treatment suggests decreased activity of PEP carboxylase [24] and consequently reduced replenishment of TCA/Krebs cycle intermediates.

Increased phosphorylation after MeJA treatment, of two non-plant specific peptides derived from Pyruvate Dehydrogenase (PDH) are associated with decreased PDH activity [25], resulting in a lower conversion of pyruvate to acetyl-CoA necessary to drive the TCA/Krebs cycle [26]. These PDH phosphorylation profiles thus point to reduced substrate feeding into the TCA/Krebs cycle in the presence of MeJA.

The light reactions of photosynthesis are mediated by photosystem I (PSI) and PSII. The PSII reaction center contains two D proteins. D proteins are phosphorylated in a circadian fashion by the specific kinase STN8 [27,28]. High light conditions increase the relative level of phosphorylated PSII D proteins [27]. The suggestion that MeJA increases phosphorylation levels of PSIID thus indicates that MeJA brings this protein in a high light state. It is to our knowledge unknown what the effect of phosphorylation of PSII D proteins is, but a role in prevention of breakdown and/or inhibition of photosynthesis is suggested [27].

Phytochrome A, one of the five red and far-red light perceiving Ser/Thr kinases (PhyA-PhyE) regulates the transcription of *psbD*, encoding the PSII D2 protein [29]. Interestingly, from our PepChips PhyA is suggested to be strongly phosphorylated upon MeJA and SA/MeJA treatment (Table 1). The annotated kinase for the PhyA-derived peptide on the PepChip is PKA [30]. In plants, the existence of PKA is unclear (see above) and the exact *in vivo* PhyA phosphorylating kinase remains therefore unknown.

**References**

1. Forsberg J, Allen JF (2001) Protein tyrosine phosphorylation in the transition to light state 2 of chloroplast thylakoids. Photosynth Res 68: 71-79.

2. Rudrabhatla P, Reddy M, Rajasekharan R (2006) Genome-Wide Analysis and Experimentation of Plant Serine/ Threonine/Tyrosine-Specific Protein Kinases. Plant Molec Biol 60: 293-319.

3. Diks SH, Parikh K, van der Sijde M, Joore J, Ritsema T et al. (2007) Evidence for a minomal eukaryotic phosphoproteome? PLoS ONE 2: e777.

4. Mayrose M, Bonshtien A, Sessa G (2004) LeMPK3 is a mitogen-activated protein kinase with dual specificity induced during tomato defense and wounding responses. J Biol Chem 279: 14819-14827.

5. Hirayama T, Oka A (1992) Novel protein kinase of Arabidopsis thaliana (APK1) that phosphorylates tyrosine, serine and threonine. Plant Mol Biol 20: 653-662.

6. Nuhse TS, Peck SC, Hirt H, Boller T (2000) Microbial elicitors induce activation and dual phosphorylation of the Arabidopsis thaliana MAPK 6. Journal of Biological Chemistry 275: 7521-7526.

7. Hwang I, Chen HC, Sheen J (2002) Two-component signal transduction pathways in Arabidopsis. Plant Physiol 129: 500-515.

8. Ferreira FJ, Kieber JJ (2005) Cytokinin signaling. Current Opinion in Plant Biology 8: 518-525.

9. Anthony RG, Henriques R, Helfer A, Meszaros T, Rios G et al. (2004) A protein kinase target of a PDK1 signalling pathway is involved in root hair growth in Arabidopsis. EMBO J 23: 572-581.

10. Gross SD, Anderson RA (1998) Casein Kinase I: spatial organization and positioning of a multifunctional protein kinase family. Cell Signal 10: 699-711.

11. Homma MK, Homma Y (2005) Regulatory role of CK2 during the progression of cell cycle. Mol Cell Biochem 274: 47-52.

12. Poole A, Poore T, Bandhakavi S, McCann RO, Hanna DE et al. (2005) A global view of CK2 function and regulation. Mol Cell Biochem 274: 163-170.

13. Allada R, Meissner RA (2005) Casein kinase 2, circadian clocks, and the flight from mutagenic light. Mol Cell Biochem 274: 141-149.

14. De Schutter K, Joubes J, Cools T, Verkest A, Corellou F et al. (2007) Arabidopsis WEE1 kinase controls cell cycle arrest in response to activation of the DNA integrity checkpoint. Plant Cell 19: 211-225.

15. Swiatek A, Azmi A, Stals H, Inze D, Van Onckelen H (2004) Jasmonic acid prevents the accumulation of cyclin B1;1 and CDK-B in synchronized tobacco BY-2 cells. FEBS Letters 572: 118-122.

16. Pauwels L, Morreel K, De Witte E, Lammertyn F, Van Montagu M et al. (2008) Mapping methyl jasmonate-mediated transcriptional reprogramming of metabolism and cell cycle progression in cultured Arabidopsis cells. Proc Natl Acad Sci USA 105: 1380-1385.

17. Johansson I, Karlsson M, Johanson U, Larsson C, Kjellbom P (2000) The role of aquaporins in cellular and whole plant water balance. Biochim Biophys Act 1465: 324-342.

18. Azad AK, Sawa Y, Ishikawa T, Shibata H (2004) Phosphorylation of plasma membrane aquaporin regulates temperature-dependent opening of tulip petals. Plant Cell Physiol 45: 608-617.

19. Eisenbarth DA, Weig AR (2005) Dynamics of aquaporins and water relations during hypocotyl elongation in Ricinus communis L. seedlings. J Exp Bot 56: 1831-1842.

20. Hrabak EM, Chan CW, Gribskov M, Harper JF, Choi JH et al. (2003) The Arabidopsis CDPK-SnRK superfamily of protein kinases. Plant Physiol 132: 666-680.

21. Guenther JF, Chanmanivone N, Galetovic MP, Wallace IS, Cobb JA et al. (2003) Phosphorylation of soybean nodulin 26 on serine 262 enhances water permeability and is regulated developmentally and by osmotic signals. Plant Cell 15: 981-991.

22. Tripodi KE, Turner WL, Gennidakis S, Plaxton WC (2005) *In vivo* regulatory phosphorylation of novel phospho*enol*pyruvate carboxylase isoforms in endosperm of developing castor oil seeds. Plant Physiol 139: 969-978.

23. Sanchez R, Flores A, Cejudo F (2006) Arabidopsis phospho enol pyruvate carboxylase genes encode immunologically unrelated polypeptides and are differentially expressed in response to drought and salt stress. Planta 223: 901-909.

24. Alvarez R, Garcia-Maurino S, Feria AB, Vidal J, Echevarria C (2003) A conserved 19-amino acid synthetic peptide from the carboxy terminus of phosphoenolpyruvate carboxylase inhibits the in vitro phosphorylation of the enzyme by the calcium-independent phosphoenolpyruvate carboxylase kinase. Plant Physiol 132: 1097-1106.

25. Thissen J, Komuniecki R (1988) Phosphorylation and inactivation of the pyruvate dehydrogenase from the anaerobic parasitic nematode, *Ascaris suum*. Stoichiometry and amino acid sequence around the phosphorylation sites. J Biol Chem 263: 19092-19097.

26. Jiao J, Vidal J, Echevarria C, Chollet R (1991) *In vivo* regulatory phosphorylation site in C4-leaf phospho*enol*pyruvate carboxylase from maize and sorgum. Plant Physiol 96: 297-301.

27. Booij-James IS, Swegle WM, Edelman M, Mattoo AK (2002) Phosphorylation of the D1 photosystem II reaction center protein is controlled by an endogenous circadian rhythm. Plant Physiol 130: 2069-2075.

28. Vainonen JP, Hansson M, Vener AV (2005) STN8 Protein Kinase in Arabidopsis thaliana Is Specific in Phosphorylation of Photosystem II Core Proteins. J Biol Chem 280: 33679-33686.

29. Thum KE, Kim M, Christopher DA, Mullet JE (2001) Cryptochrome 1, Cryptochrome 2, and Phytochrome A Co-Activate the Chloroplast psbD Blue Light-Responsive Promoter. Plant Cell 13: 2747-2760.

30. McMichael RWJ, Lagarias JC (1990) Phosphopeptide mapping of *Avena* phytochrome phosphorylated by protein kinases in vitro. Biochem 29: 3872-3878.
